# Recent Advances and Retrospective Review in Bioinspired Structures for Fog Water Collection

**DOI:** 10.3390/biomimetics10120791

**Published:** 2025-11-21

**Authors:** Shizhang Dong, Guangze Li, Shaobo Jin, Hong Hu, Guoyong Ye

**Affiliations:** 1 Henan Key Laboratory of Intelligent Manufacturing Mechanical Equipment, Zhengzhou University of Light Industry, Zhengzhou 450000, China; 2 School of Microelectronics, Shanghai University, Shanghai 201800, China; 3 Sino-Swiss Institute of Advanced Technology (SSIAT), Shanghai University, Shanghai 201899, China

**Keywords:** fog water collection, biomimetic structures, surface wettability, directional droplet transport

## Abstract

Fog water collection, as a sustainable approach to alleviating water scarcity, has attracted considerable attention due to its low energy consumption and environmental friendliness. Various organisms in nature have evolved unique biological structures that efficiently capture and direct fog water. The fog water collection structures (FWCSs) and physical mechanisms of these organisms provide valuable inspiration for innovations in fog water collection technologies. This review systematically summarizes biomimetic structures designed for fog water collection, with a focus on representative natural examples such as the Namib desert beetle, cactus spines, spider silk, and *Nepenthes mirabilis*, highlighting how they achieve efficient fog water capture, coalescence, and transport through special surface textures, wettability regulation, and structural design. The underlying physical mechanisms are discussed in depth, including droplet behavior on micro/nanostructured surfaces, surface energy gradients, and Laplace pressure gradients in directional droplet transport. On this basis, the current challenges in bioinspired FWCSs design are outlined, and future perspectives are proposed. Future research may focus on the multiscale structural optimization of bioinspired FWCSs, the development of dynamically tunable designs, and the use of efficient and sustainable materials to further enhance fog water collection efficiency and ensure the long-term stability of FWCSs. Ultimately, by integrating modern manufacturing technologies and stimuli-responsive materials, bioinspired FWCSs hold great potential for applications in extreme environments, agricultural irrigation, and energy-efficient architecture, offering innovative solutions to the global water crisis.

## 1. Introduction

Water is an indispensable and valuable resource for human production, daily life, and social services, with drinking water being essential for human survival [1]. The problem of global water scarcity is not only widespread in deserts, semi-deserts, coastal regions, islands, and mountainous areas, but has also become increasingly prominent in other regions [2]. The causes of growing water shortages are complex and multifaceted. First, the uneven distribution of water resources leads to severe shortages in certain regions. Second, climate change has intensified desertification processes in some areas, causing rivers to dry up. Third, the rapid economic, social, and industrial development has driven a continuous increase in demand for water in industry, agriculture, and the service sector. Fourth, population growth, environmental pollution, and the overexploitation of groundwater have together exacerbated structural water shortages [3].

To address the challenges of water scarcity and more effectively meet the growing demand for drinking water, researchers have actively investigated various technologies for water-harvesting from the natural environment, including seawater desalination [4], rainwater harvesting [5], artificial precipitation [6], atmospheric water extraction, and fog water collection [7]. At present, the primary method of freshwater acquisition is seawater desalination, which removes salts from seawater through distillation, reverse osmosis, chemical methods, or freezing to produce potable water [8]. Although feasible in water-scarce regions, this approach is costly and thus difficult to implement in impoverished areas suffering from extreme water shortages. Rainwater, as a natural source of freshwater, can be collected and stored for human use through drainage systems installed on rooftops that channel water into storage tanks or barrels. However, this method is impractical in arid and low-rainfall regions [9]. Artificial precipitation and atmospheric water extraction generally require large-scale equipment, making them more expensive compared with fog water collection technologies.

Fog is an aerosol system composed of tiny, visible fog droplets suspended near the ground, with fog droplet sizes ranging from 1 to 40 μm [10]. Fog water collection is a technique that captures and harvests liquid water by intercepting, capturing, and channeling these fog droplets through specially designed fog water collection structures (FWCSs) or surface materials [11]. Owing to its simplicity, environmental friendliness (no external energy input), and low operation and maintenance costs, this technique is regarded as a highly promising and sustainable approach for obtaining water in specific arid regions such as coastal areas, mountains, and desert margins. The working principle of fog water collection is that, under natural wind conditions with speeds of approximately 2–10 m/s, fog droplets collide with the surface of collection materials, enabling the interception of fog droplets with sizes typically ranging from 1 to 12 μm. These fog droplets subsequently coalesce into larger droplets or continuous streams, which are directed into collection troughs or storage tanks via designed channels [12]. The feasibility and practical value of fog water collection have already been well demonstrated [13,14].

Over the past decade, more than 17 countries worldwide have successfully implemented fog water collection projects [15]. The Food and Agriculture Organization of the United Nations (FAO) highlighted in a recent report that “fog water collection is a highly promising and low-cost water acquisition mechanism suitable for drinking water supply, agricultural irrigation, and livestock farming in arid regions” [16]. Most of these projects employ fiber-based mesh structures, which capture fog droplets through interactions between the mesh surface and the fog flow field, allowing the droplets to deposit on the mesh and be harvested [17,18]. By tailoring the geometry of the mesh openings, the aerodynamic characteristics of the fog water collection process can be effectively improved, thereby enhancing collection performance [19]. However, conventional mesh-FWCSs still face significant limitations: their collection efficiency remains relatively low, their long-term efficiency is difficult to maintain, and numerous practical challenges persist in field applications. For example, Maher Damak and Kripa K. Varanasi [20] attempted to enhance droplet deposition efficiency on metal meshes by applying an external electric field.

While this approach overcomes one of the major shortcomings of traditional meshes—where a large fraction of droplets either penetrate the openings or bypass the grid without being captured—it also presents non-negligible drawbacks. Specifically, such fog water collection systems are structurally complex, difficult to install, dependent on external power sources (potentially introducing environmental concerns), and challenging to scale up large-area deployment. Moreover, under adverse conditions such as strong winds, the intense wind pressure may not only tear the mesh surface but also topple the supporting framework of the fog water collection system.

**Figure 1:**
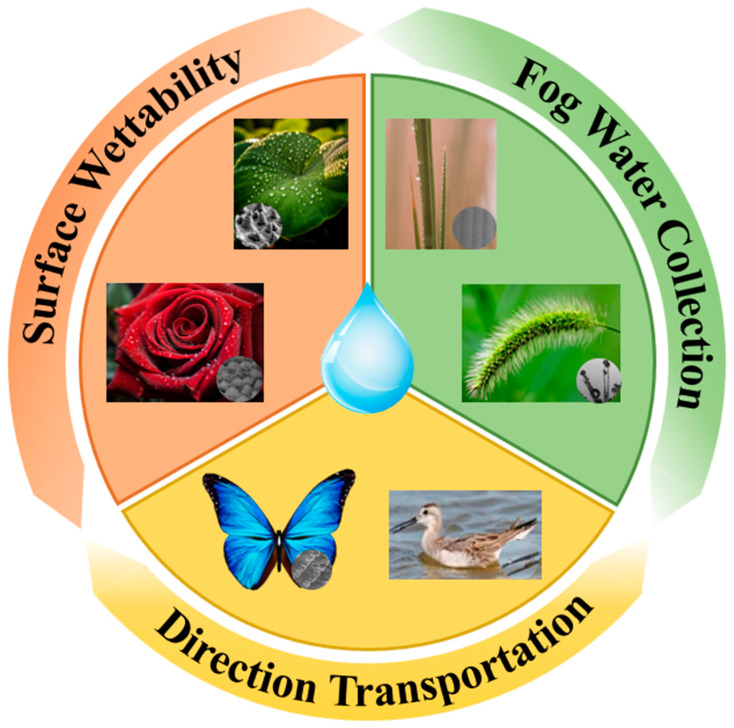
Plants and animals in nature with fog water collection structures. Lotus Leaf (Reprinted with permission from ref. [22], Copyright ©2015 Elsevier Ltd. All rights reserved.), Rose Petal (Reprinted with permission from ref. [23], Copyright ©2008, American Chemical Society), Stipagrostis sabulicola (Namibian needle grass) (Reprinted with permission from ref. [24], Copyright ©2004, Royal Society), Foxtail Grass (Reprinted with permission from ref. [35], Copyright ©2011, RSC Publishing), Butterfly (Reprinted with permission from ref. [36], Copyright ©2005, Royal Society of Chemistry), Shorebird (Reprinted with permission from ref. [37], Copyright ©2008, The American Association for the Advancement of Science).

Nature has long served as a rich source of inspiration for technological innovation, and its ingenuity is vividly embodied in organisms thriving in arid deserts. Many plants and animals have evolved intricate body structures that enable them to capture water from fog flows with remarkable efficiency [21]. In recent years, bioinspired structures for fog water collection have attracted growing attention, drawing inspiration from natural models such as lotus leaves [22], rose petals [23], the Namib desert grass *Stipagrostis sabulicola* [24] (Figure 1 and Table 1), and the animals like the Namib desert beetle and the Australian thorny devil (*Moloch horridus*) [25], and this line of research has been increasingly highlighted in recent studies [26,27]. Although each species exhibits distinct fog water collection mechanisms (for example, the thorny devil captures fog water through capillary channels on its back [28], which differs somewhat from the four representative models reviewed in this paper), all of them provide indispensable design strategies for the development of fog water collection structures (FWCSs). Meanwhile, by analyzing the fog water collection surfaces and observing the collection behaviors of these fascinating organisms in nature, significant progress has also been made in the design and fabrication of bioinspired FWCSs. Researchers have identified several key features associated with fog water collection, including micro-textures, special wettability, and hierarchical architectures [29]. Based on these findings, a series of fog water collection structures incorporating bioinspired materials and tailored wettability have been designed and fabricated to enhance fog water collection efficiency [30,31]. To date, several bioinspired mechanisms have been proposed and validated for their effectiveness in improving fog water capture, and multiple strategies for designing and fabricating wettability-engineered surfaces for fog water collection have been developed [32,33]. These advances in bioinspired structures and materials hold great promise for enhancing freshwater availability in arid regions [34].

**Figure 2:**
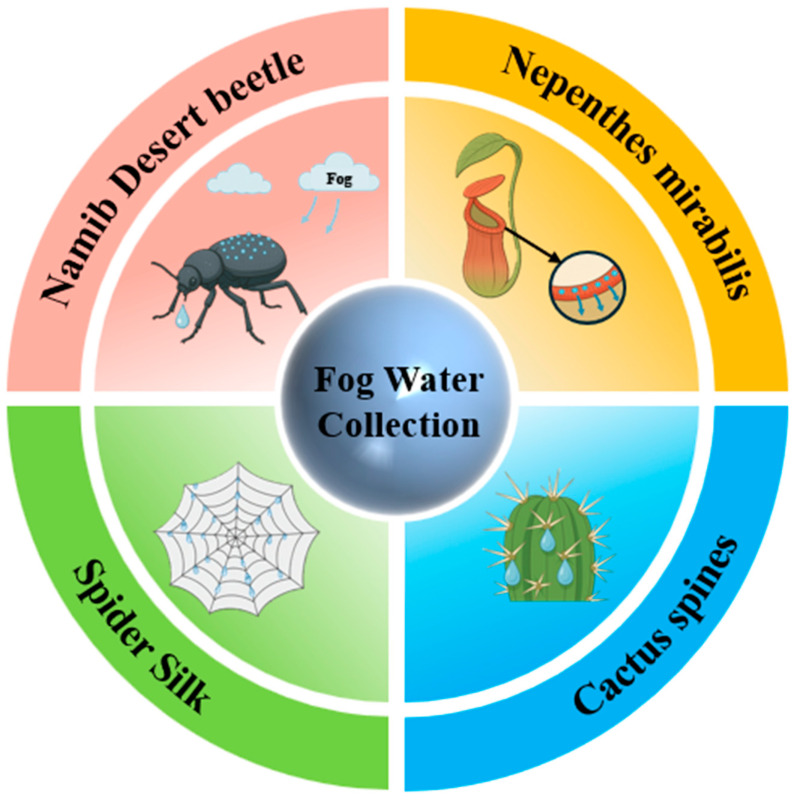
Four representative biological prototypes for fog water collection structures. Namib Desert beetle, Cactus Spines, Spider Silk, Nepenthes mirabilis.

In this review, we systematically summarize recent progress in bioinspired fog water collection, with a particular emphasis on its theoretical foundations and key structural designs. The discussion is organized around four essential stages of the fog water collection process—fog water capture and condensation, coalescence, directional transport, and absorption—each elucidated from a mechanistic perspective. Furthermore, optimization strategies inspired by four representative biological prototypes—the Namib desert beetle, cactus spines, spider silk, and *Nepenthes mirabilis*—are analyzed in detail (as shown in Figure 2). Finally, the current research status is summarized, and future perspectives on the development of bioinspired FWCSs are presented.

## 2. Theoretical Basis of Fog Water Collection

Fog water collection involves four key processes: fog water capture and condensation, coalescence, directional transport, and absorption. Fog water capture and condensation are influenced by surface wettability and aerodynamics. Once captured, the static and dynamic behaviors of microdroplets on the surface are affected by the gradient forces of FWCSs and the surface wettability of the material. In other words, the processes of microdroplets coalescence, directional transport, and absorption at specific sites are governed by Laplace pressure gradients and surface energy gradients. These theoretical models help clarify the mechanisms of fog water collection by explaining the static and dynamic interactions of droplets on surfaces, thereby providing a solid foundation for further analysis of microdroplets coalescence, motion, and the directional transport of coalesced droplets on the material surfaces of FWCSs.

### 2.1. Surface Wettability and Contact Angle

Liquid wetting on solid surfaces is a common interfacial phenomenon and an important characteristic of solid surfaces [42]. The coalescence and directional transport of microdroplets condensed on fog water collection structures are inseparable from the properties of the underlying surface. Young’s equation and the contact angle are used to quantify the degree of wetting of a liquid on a solid.

#### 2.1.1. Young’s Equation

**Figure 3:**
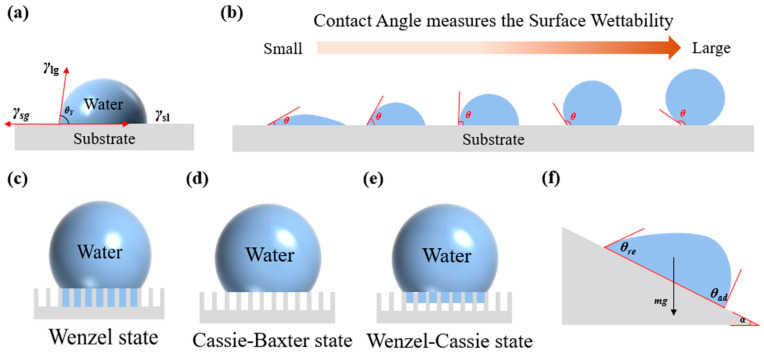
Schematic illustration of droplets on solid surfaces. (**a**) Droplet on an ideal smooth surface (Young’s equation). (**b**) Contact angle as an indicator of surface wettability. (**c**) Droplet on a fully wet rough surface in the Wenzel state. (**d**) Droplet on a rough surface in the Cassie–Baxter state, where air cavities prevent complete wetting. (**e**) Droplet in the combined Wenzel–Cassie state. (**f**) Contact angle hysteresis: when the advancing angle (*θ*_ad_) and receding angle (*θ*_re_) reach the hysteresis threshold, the droplet begins to move.

Surface wettability reflects the ability or tendency of a droplet to move on a surface. When a droplet spreads on a solid surface, three interfaces (with corresponding surface tensions) are formed: solid–gas (*γ*_sg_), solid–liquid (*γ*_sl_), and liquid–gas (*γ*_lg_). The line where these three phases meet is called the three-phase contact line. Interfacial tension (e.g., *γ*_sg_, *γ*_sl_, *γ*_lg_) can be understood as the force per unit length acting along this line, numerically equal to the interfacial energy per unit area. The contact angle is a key parameter for evaluating wettability, defined as the angle formed between the tangent to the liquid–gas interface and the solid–liquid interface at the three-phase boundary [43,44]. The magnitude of the contact angle results from the combined action of the three interfacial tensions. As shown in Figure 3a, in 1805 Thomas Young described the relationship between interfacial tensions and the contact angle on an ideal, flat, chemically homogeneous surface [45]:

(1) $$\begin{array}{c} \gamma_{\mathrm{sg}}=\gamma_{\mathrm{sl}}+\gamma_{\mathrm{lg}}\times \cos\theta_{Y} \end{array}$$

That is,

(2) $$\cos\begin{array}{c} \theta_{Y}=\frac{\gamma_{\mathrm{sg}}-\gamma_{\mathrm{sl}}}{\gamma_{\mathrm{lg}}} \end{array}$$

Here, *θ_Y_* is the equilibrium contact angle, also referred to as the intrinsic contact angle of the material. As shown in Figure 3b, Equation (2) can be used to predict different wetting states: (1) *θ_Y_* = 0, complete wetting; (2) *θ_Y_* < 90°, hydrophilic, partial wetting; (3) *θ_Y_* = 90°, the boundary between hydrophilicity and hydrophobicity; (4) *θ_Y_* > 90°, hydrophobic, non-wetting; (5) *θ_Y_* = 180°, completely non-wetting. Therefore, based on Young’s equation, the contact angle can be calculated to determine whether a material surface is hydrophilic or hydrophobic.

For the same liquid, *γ*_sl_ and *γ*_lg_ usually do not vary significantly; thus, the contact angle is largely determined by *γ*_sg_ [46]. The balance among the three surface tensions determines the wetting state of the droplet. When a droplet rests on the surface, the three surface tensions tend to reduce the interfacial area by controlling the contact line. Eventually, the three surface tensions reach equilibrium, and the droplet maintains a stable or metastable state on the surface [47].

Young’s equation is formulated under the assumption that a non-reactive liquid (chemically and physically inert) spreads on an ideal solid surface that is smooth, homogeneous, and rigid. In addition, it assumes that the contact angle is sufficiently large to allow accurate measurement, a condition that is rarely satisfied in practice. Young’s equation serves as the most fundamental starting point for understanding the wetting behavior of complex solid–liquid interfaces. Moreover, the roughness of the material surface is another important parameter affecting droplet–surface interactions.

#### 2.1.2. Wenzel State

In real-world situations, material surfaces are rarely perfectly smooth, and the wettability of droplets on rough surfaces must be considered. Because of surface tension, the actual solid–liquid interfacial contact area is larger than that of an ideal flat surface, resulting in a difference between the apparent contact angle and the ideal contact angle. Wenzel [48,49] proposed that droplets can fill the grooves of a rough surface (Figure 3c), thereby modifying Young’s equation. At equilibrium, the contact angle of a droplet on a rough surface is expressed as:

(3) $$\begin{array}{c} \cos\theta_{r}=r\cos\theta_{Y} \end{array}$$

where *θ*_r_ is the apparent contact angle, *θ_Y_* is the intrinsic contact angle, and *r* is the surface roughness (the ratio of the actual solid–liquid interfacial area to the apparent area), which is always greater than 1. According to Equation (3), when the intrinsic contact angle is less than 90°, increasing surface roughness decreases the apparent contact angle, meaning that rougher hydrophilic surfaces become more hydrophilic. Conversely, when the intrinsic contact angle is greater than 90°, increasing surface roughness increases the apparent contact angle, meaning that rougher hydrophobic surfaces become more hydrophobic. Thus, by altering the roughness of the surface, the apparent contact angle of droplets on the surface can be regulated, thereby modifying the wettability of the surface.

The Wenzel equation applies only to surfaces with uniform chemical composition and roughness. Because droplets in the Wenzel state are completely anchored within the rough structure, the liquid typically exhibits high adhesion and is difficult to displace.

#### 2.1.3. Cassie–Baxter State

Building on the Wenzel state, Cassie and Baxter [50] further extended and modified Young’s equation. They proposed that a droplet on a rough surface exhibits composite contact. Specifically, when a droplet cannot wet the surface asperities, air becomes trapped within the grooves, forming an “air cushion”. The droplet thus rests on a composite surface consisting of both solid and air. This assumption is closer to real conditions: when the surface roughness is sufficiently heterogeneous, liquid can easily trap air pockets within the grooves. Consequently, part of the droplet base contacts the rough solid surface, forming a liquid–solid interface, while the other part contacts air, forming a liquid–air interface (Figure 3d). The apparent contact angle in this case is given by:

(4) $$\begin{array}{c} \cos\theta_{r}=f\cos\theta_{Y}+f \end{array}-1$$

where *f* represents the ratio of the liquid–solid interfacial area. For hydrophobic surfaces, a smaller *f* leads to a larger apparent contact angle. The Cassie–Baxter equation effectively explains superhydrophobicity: the air cushion reduces wetting of the solid surface, allowing droplets to roll off easily. Therefore, the Cassie–Baxter state is characterized by low adhesion.

From Equations (3) and (4), it follows that the apparent contact angle depends on both the intrinsic contact angle and the surface microstructure. Thus, the wetting behavior of a droplet is governed jointly by surface chemistry and roughness, laying the theoretical foundation for investigating droplet dynamics.

#### 2.1.4. Wenzel–Cassie State

The Wenzel and Cassie wetting state describe two extreme states of a droplet on a rough solid surface. In practice, however, external environmental factors also influence the wetting state of liquids. For example, during compression or impact, a droplet may gradually transition from the Cassie–Baxter state to the Wenzel state. Part of the droplet penetrates into the air cushion of the surface rough structure, while another part remains on the air cushion, resulting in a mixed wetting state (Figure 3e). To better explain this complex wetting behavior, Rios et al. proposed a more comprehensive and accurate wetting equation [51]:

(5) $$\sin a=\frac{K\pi f}{g}{\left[\frac{3}{\rho \pi (2-3\cos\theta_{r}+\cos^{3}\theta_{r})}\right]}^{2/3}\sin^{2}\theta_{r}m^{1/3}$$

where *α* represents the sliding angle of the liquid on the rough surface; *K* is the interfacial adhesion parameter related to the surface energy of the solid; g is gravitational acceleration; *ρ* is the liquid density; and *m* is the liquid mass. In this state, droplets typically exhibit stronger adhesion, more pronounced contact angle hysteresis, and an increased sliding angle.

#### 2.1.5. Contact Angle Hysteresis

On an ideal smooth horizontal surface, a droplet can maintain a stable static contact angle. When a small volume of liquid is added to or removed from the droplet, only its perimeter changes, while the contact angle on the surface remains constant. Although the contact angle is a key indicator of a solid surface’s hydrophobicity, assessing superhydrophobic performance also requires consideration of the droplet’s dynamic behavior [52].

When the solid surface is tilted so that the droplet is on the verge of rolling but does not yet roll—that is, at the critical rolling state—the angle between the tilted surface and the horizontal line is denoted as *α*. At this point, the droplet exhibits two distinct contact angles: the advancing angle (*θ_ad_*) and the receding angle (*θ_re_*), as shown in Figure 3f. The advancing angle refers to the contact angle at which the three-phase contact line is about to move, but has not yet moved, when the droplet volume increases. It can be understood as the angle the front slope of the droplet must reach for sliding to occur. Conversely, the receding angle is the contact angle at which the three-phase contact line is about to move, but has not yet moved, when the droplet volume decreases. It can be understood as the angle the rear slope of the droplet must reduce to in order for sliding to occur [53].

In general, the advancing angle is larger than the receding angle, and their difference (*θ_ad_* − *θ_re_*) is defined as the contact angle hysteresis of the droplet [54]. The greater the difference between *θ_ad_* and *θ_re_*, the less likely the droplet is to detach from the solid surface; the smaller the difference, the more easily the droplet detaches. The sliding angle *α* characterizes the degree of contact angle hysteresis on rough solid surfaces (Figure 3f). The contact angle hysteresis arises because of an energy barrier at the droplet’s leading edge [55]. In 1962, Furmidge proposed a relationship between contact angle hysteresis and sliding angle to explain the retention of droplets on inclined surfaces. This relationship is expressed by the Furmidge equation, as shown in Equation (6) [56,57]:

(6) $$mg\sin\alpha =\gamma_{\mathrm{lg}}(\cos\theta_{re}-\cos\theta_{ad})w$$

In the equation, *α*, *θ_re_*, and *θ_ad_* represent the sliding angle, receding angle, and advancing angle of the droplet, respectively. *m* is the droplet’s mass, *g* is the gravitational acceleration, *γ_lg_* is the surface tension of the droplet, and *w* is the width of the droplet. The contact angle hysteresis of the droplet is considered the primary barrier to its movement on the surface. This phenomenon occurs when a droplet on a rough or uneven solid surface, as long as the lateral adhesion between the droplet and the solid allows, exhibits a metastable contact angle (Figure 3f). The rough surface fixes the three-phase contact line of the droplet to the substrate. When the droplet overcomes static friction, the contact line moves first, and the force applied to the droplet does not directly cause it to move, but instead deforms the droplet at a specific contact angle. The droplet will only move once a certain threshold is reached.

The sliding angle significantly impacts the application of superhydrophobic surfaces. Only superhydrophobic surfaces with a small sliding angle (*α* less than 5°) exhibit characteristics such as self-cleaning and anti-adhesion, making them suitable for a wide range of applications. These surfaces are commonly referred to as the Lotus state (Lotus effect). High adhesion superhydrophobic surfaces differ considerably from typical superhydrophobic surfaces in terms of sliding angle. On these surfaces, a droplet can flip 180° without falling off, a phenomenon known as the Petal state (Petal effect) [58]. Based on the sliding angle of superhydrophobic surfaces, they are typically classified by surface adhesion strength, ranging from Lotus state, Cassie state, Cassie-Wenzel transition state, Wenzel state, to Petal state. The adhesion strength of superhydrophobic surfaces is determined by both surface morphology and material composition. Non-uniform surface chemical composition or morphology can increase surface adhesion strength [59].

### 2.2. Fog Water Capture and Aerodynamic Factors

Fog droplets are commonly visible in nature, with their density varying depending on the source. The size and distribution of these fog droplets are crucial factors in determining fog water collection efficiency. Larger fog droplets, although less common in the airflow, deposit more quickly; by contrast, smaller fog droplets maintain the stability of the fog flow but deposit more slowly. In fog-prone regions, fog droplets carried by the wind are frequently intercepted by vegetation surfaces, where they gradually coalesce, enlarge, and eventually roll off the leaves onto the ground. Because static fog is difficult to capture efficiently, the dynamic behavior of fog flow is critical for effective collection.

In theory, the performance of FWCSs inspired by organisms in nature is determined by their ability to capture fog water and direct their transport. Fog is first intercepted on the surface of the FWCSs, then guided through the designed channels toward the collection point, where the surface is refreshed to enable the next cycle of fog water capture. Fog water capture represents the initial stage of interaction between the structure of FWCSs and the fog flow, and this process can be explained by two distinct physical mechanisms: (1) collision and deposition of small fog droplets without phase change, and (2) water vapor condensation and deposition with phase change [60]. Understanding the differences and coupling between these two mechanisms forms the theoretical basis for designing efficient bioinspired FWCSs.

#### 2.2.1. Collision and Deposition of Small Fog Droplets Without Phase Change

The collision and deposition of small fog droplets without phase change is essentially the interaction between the fog flow and the surface of the fog water collection structure. When the relative humidity is high, liquid small fog droplets may coalesce in the fog flow. Driven by wind, these fog droplets move along with the fog flow. As the fog flow passes over the surface of the fog water collection structure, the fog droplets deviate from their flow paths due to inertia and collide with the surface of the structure. The surface of the structure acts as an obstacle, causing a portion of the small fog droplets to deposit onto the surface, thereby being captured. The fog droplets deposited on the conical surface gradually grow into small liquid droplets. At the same time, small fog droplets in the fog collide with small liquid droplets, causing adjacent droplets to coalesce and increase in volume, forming larger water droplets.

The liquid droplets captured by the surface of the FWCSs through the collision and deposition of small fog droplets without phase change are referred to as the fog interception effect (the effective interception efficiency of the fog flow) [61]. Langmuir and Blodgett [62] proposed an empirical formula for the collision and deposition of small droplets on a surface with curvature *K*. Specifically, the deposition efficiency *ηd* of small droplets can be approximately calculated using the empirical formula [63,64]:

(7) $$\eta_{d}\approx 1-\frac{2}{Ku_{0}t_{0}+\pi }$$

where *u*_0_ is the fluid velocity of the fog flow, and *t*_0_ is the relaxation time of the small droplets. The deposition efficiency *η_d_* is influenced by both aerodynamic factors and the radius of curvature *K* of the surface of the fog water collection structure. However, it is important to note that different FWCSs may result in varying forms of deposition efficiency *η_d_*, but these variations are not significant, and only a correction factor is needed.

Due to the limited fog water collection area of the FWCSs, only a portion of the surface can capture fog droplets. Therefore, the shadow coefficient (*SC*) of the FWCSs defines the ratio of the effective interception area of the fog flow [65]. It is important to note that not all small fog droplets that collide with the surface will be captured; some fog droplets may deviate from their trajectories upon impact and rebound back into the fog flow. The aerodynamic collection efficiency (*η_a_*) is determined by the combined effects of the shadow coefficient (*SC*) and the fog interception effect, representing the proportion of small fog droplets that collide with the surface of the fog water collection structure [66]. The aerodynamic collection efficiency (*η_a_*) can be approximated as the product of the following functions [67]:

(8) $$\eta_{a}=\frac{SC}{1+\sqrt{C_{o}/C_{d}}}$$

where *C*_o_ is the pressure drop coefficient of the FWCSs, and *C*_d_ is the resistance coefficient of the FWCSs, which varies depending on the shape of the FWCSs. The pressure drop coefficient *C*_o_ can be expressed as [68]:

(9) C0=KRe1.3SC+SC1−SC2

where *K*_Re_ is a correction factor dependent on fluid properties (e.g., Reynolds number).

#### 2.2.2. Water Vapor Condensation and Deposition with Phase Change

On the other hand, the condensation process involving a phase change refers to the gas–liquid transformation of water droplets on the surface of FWCSs. The initial step of condensation is the nucleation of the smallest droplets on a solid surface at ambient temperature [69]. These small droplets subsequently coalesce into larger droplets [70]. The efficiency of gas–liquid phase change in droplets on the surface of FWCSs is governed by the relationship between the saturation vapor pressure and the actual vapor pressure [71]. According to the Kelvin equation [72] (Equation (10)), the actual vapor pressure *P* at the gas–liquid interface of droplets can be calculated as:

(10) $$P=e^{\frac{2\gamma V_{\mathrm{ml}}}{RT}K}P_{s}$$

where *R* is the ideal gas constant, *T* is the ambient temperature, *γ* is the surface tension of droplet (approximately constant), *V_ml_* is the molar volume of droplet, *P_s_* is the saturation vapor pressure of droplet on a smooth surface, and *K* is the surface curvature of the captured droplet. Accordingly, the critical condensation radius (*r_c_*) of droplet can be derived as [73]:

(11) $$r_{c}=\frac{1}{K}=\frac{2\gamma V_{\mathrm{ml}}}{\left[RT\ln(P/P_{s})\right]}$$

At the initial stage of droplet condensation, the droplet surface curvature (*K*) can be approximated by the curvature of the solid–liquid interface. Increasing the solid–liquid interfacial curvature raises the vapor pressure (*P*) and decreases the critical condensation radius (*r_c_*), thereby accelerating vapor condensation from fog. Equation (11) also indicates that reducing surface tension lowers the critical condensation radius. Some studies have suggested that, because droplets on hydrophobic surfaces exhibit a lower effective surface tension than those on hydrophilic surfaces, the critical condensation size on hydrophobic surfaces is smaller. However, this interpretation is not entirely accurate. In Equation (11), *γ* refers to the intrinsic surface tension of water, which is generally constant. Although surface tension directly affects *r_c_*, the actual condensation behavior is primarily dictated by surface wettability. On hydrophobic surfaces, the high contact angle and low surface energy substantially increase the nucleation barrier for small droplets, despite the apparent reduction in *r_c_*. By contrast, hydrophilic surfaces promote spontaneous adsorption and coalescence of small droplets into continuous liquid films, markedly lowering the nucleation barrier and ultimately facilitating condensation more effectively [74].

A relatively high condensation density on the surface of fog water collection structures is a prerequisite for efficient condensation. Studies have shown that condensation density is higher on hydrophilic surfaces than on hydrophobic ones; therefore, in the design of FWCSs, hydrophilic surfaces are often employed as the primary condensation interface. The condensation density on hydrophilic surfaces can be tuned by adjusting the density of hydrophilic sites, and compared with superhydrophobic surfaces, the fog water collection efficiency of hydrophilic surfaces can be up to 349% higher [75]. In addition to condensation, droplet growth can also be artificially regulated. During the condensation process, a pronounced edge effect can be observed: droplets located at corners or edges grow faster than those on flat regions, as they can capture more water vapor during condensation. In some cases, this growth enhancement can reach as high as 500% [76,77]. Since water vapor condensation requires a number of specific conditions, research on fog water collection primarily focuses on capturing pre-existing fog droplets suspended in the air. In this process, hydrophilic sites exhibit stronger fog water collection capability than superhydrophobic ones.

#### 2.2.3. Filmwise and Dropwise Condensation in Fog Water Collection

During phase change condensation of water vapor, heat transfer is inevitable. The faster the heat transfer, the faster droplets condense on the surface of the fog water collection structures, thereby improving overall collection efficiency. In the fog water collection process, condensed microdroplets on the surface sometimes coalesce into a continuous liquid film that completely wets the surface of the FWCSs. As the liquid film accumulates and thickens, it flows downward along the surface of the FWCSs under gravity, a process referred to as filmwise condensation collection. In contrast, when condensation does not fully wet the surface and water remains in the form of discrete droplets adhering to the surface, the process is called dropwise condensation collection. Because filmwise condensation collection maintains a continuous liquid layer covering the surface, phase change heat transfer between water vapor and the surface must occur through the liquid film. Given that the thermal conductivity of the film is very low, this significantly hinders heat transfer, making the efficiency of filmwise condensation collection generally lower than that of dropwise condensation collection.

(1) Filmwise Condensation Collection

**Figure 4:**
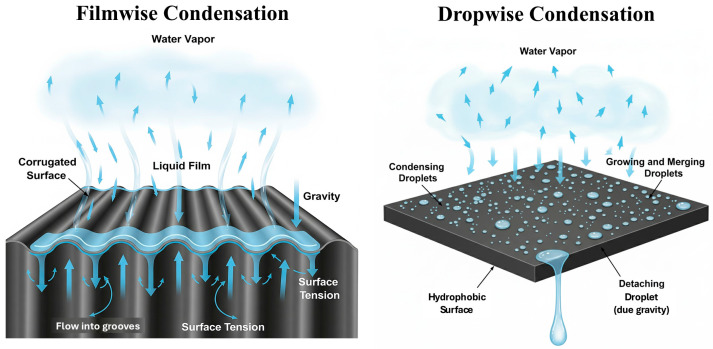
Schematic illustration of filmwise condensation collection and dropwise condensation collection.

In 1916, Nusselt proposed a heat transfer model for filmwise condensation collection [78]. This model assumes that the surface energy of the FWCSs is much greater than that of the droplets. When a liquid film can wet the surface of the fog water collection structures, the model can be applied to describe the flow state of droplets on the surface and to calculate heat transfer during the phase change condensation process. In practice, however, when the contact angle of droplets on the surface of FWCSs is relatively large—that is, when the surface exhibits hydrophobicity—the structure cannot be completely wetted, leading to deviations between the Nusselt model and real scenarios. To address this limitation, Gregorig [79] proposed a method to enhance filmwise condensation heat transfer on corrugated surfaces by utilizing liquid surface tension. The central idea of this approach is to reduce the thickness of the liquid film while increasing its heat transfer area, thereby decreasing thermal resistance. By altering the morphology of liquid flow on the surface of the fog water collection structure, the liquid film acquires different flow characteristics. As shown in Figure 4, under the influence of surface tension, the protruding parts of the collection surface remain exposed to vapor, where condensation occurs. Driven by both surface tension and gravity, droplets then flow into the grooves, forming flow channels that facilitate liquid transport.

(2) Dropwise Condensation Collection

When the surface of the FWCSs is hydrophobic, condensed water vapor is unable to form a continuous liquid film that fully wets the surface. Instead, it remains as small droplets, which continue to grow in size. Once these small droplets reach a critical size, they merge with neighboring droplets, and upon reaching another critical threshold, they slide off the surface under the influence of gravity. This detachment refreshes the FWCSs surface, initiating a new collection cycle. Because the droplets in dropwise condensation collection have relatively small contact areas with the FWCSs surface, the surface is not completely covered during the shedding process. Consequently, the heat transfer efficiency of dropwise condensation collection is generally more than five times higher than that of filmwise condensation collection.

The key factors influencing the dropwise condensation collection process include: ① the minimum radius of droplets condensed from water vapor on the surface of the FWCSs, as the condensation rate of droplets must exceed their evaporation rate; otherwise, stable microdroplets cannot form; ② the maximum radius that a single droplet can reach through self-growth, beyond which further growth occurs only by coalescence; ③ the droplets diameter at shedding, where a higher contact angle and smaller contact angle hysteresis result in smaller shedding diameters.

Studies by Glicksma et al. [80] and Graham et al. [81] demonstrated that, during dropwise condensation collection, large diameter droplets occupy up to 60% of the FWCSs surface area but contribute less than 10% of the total heat transfer, leading to low fog water collection efficiency. In contrast, droplets with diameters below 10 μm, which have smaller contact areas with the FWCS surface, account for as much as 90% of the heat transfer. Thus, the fog water collection efficiency is closely related to the droplet–surface contact area, and smaller contact areas can significantly enhance performance. Accordingly, researchers have developed superhydrophobic surfaces to minimize the contact area of droplet–FWCSs and thereby improve the efficiency of dropwise condensation collection.

Filmwise condensation collection is characterized by high surface coverage and a continuous interface. Although its heat transfer resistance is relatively high, it enables stable drainage and high-flux water transport. By contrast, dropwise condensation collection reduces heat transfer resistance through frequent droplets shedding and continuous surface “refreshing”, which facilitates rapid collection–release cycles for fog water collection.

#### 2.2.4. Overall Efficiency of Fog Water Collection

From the two primary fog water capture mechanisms, it is evident that enhancing fog water collection efficiency requires maximizing the interaction between FWCSs surfaces and fog flow. However, in practical scenarios, a substantial portion of fog droplets often pass through the FWCSs and drift away with the fog flow. Thus, increasing the effective contact area between FWCSs and fog flow is essential for improving collection efficiency.

Since fog is a two-phase fluid with complex fog droplets dynamics, the overall efficiency of fog water collection (*η*) depends not only on the aerodynamic collection efficiency (*η_a_*) but also on the deposition efficiency (*η_d_*) and drainage efficiency (*η_dr_*) [82]. Among these, the drainage efficiency *η_dr_* refers to the proportion of water successfully transported by the FWCSs to the designated collection site. Some of the captured fog water may be lost during transport due to evaporation or overflow [83]. In most numerical simulations, *η_dr_* is commonly assumed to be 1. Nevertheless, *η_dr_* can be improved by reducing flow resistance within the transport channels (e.g., by employing superhydrophilic channels), extending the fog water capture pathways, and performing fog water collection under high relative humidity conditions can effectively minimize evaporative loss and thereby enhance drainage efficiency (*η_dr_*) [84].

In summary, due to factors such as fog flow by passing the FWCSs, droplets rebounding into the fog flow after colliding with the surface, and losses during the transport of collected water, the maximum fog water collection efficiency (*η*) can be expressed by Equation (12):

(12) $$\eta =\eta_{a}\times \eta_{d}\times \eta_{dr}$$

In addition to these factors, the overall efficiency of fog water collection (*η*) is also indirectly influenced by environmental conditions, including fog flow direction, temperature, relative humidity, fog density, and droplet diameter [85]. Furthermore, the materials used, and the structural characteristics of fog water collection structures (FWCSs) play a critical role in determining overall efficiency.

### 2.3. Surface Energy Gradient and Laplace Pressure Gradient

In recent years, researchers have observed plants and animals with fog water collection capabilities, analyzing how their structural features contribute to fog water collection. Inspired by these natural models, corresponding structures have been designed, fabricated, and optimized. For some designs, sustainable fog water collection requires transferring the freshly captured fog water so that additional microdroplets can continue to be collected on the surface. During the transfer of droplets, gravitational effects are generally negligible; instead, the droplets are primarily driven by the surface energy gradient and the Laplace pressure gradient.

#### 2.3.1. Surface Energy Gradient

**Figure 5:**
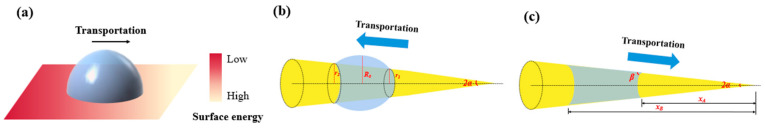
Forces acting on droplets during directional transport. (**a**) Effect of surface energy gradient. Effect of Laplace pressure gradient on (**b**) the outer surface and (**c**) the internal surface of a conical structure.

When a droplet rests on a surface with uniform chemical properties and roughness, it wets the surface equally in all directions, and the interfacial energy *U_γ_* of the solid–liquid–gas system remains constant. However, when the droplet is located on a surface with a gradient (Figure 5a), the interfacial energy *U_γ_* and the values of the three interfacial tensions change, generating a driving force *F*_D_ in the process [86]. Regions with higher surface energy promote greater droplet spreading; therefore, the driving force *F*_D_ arising from the surface energy gradient is directed toward the region of higher surface energy, with its magnitude given by:

(13) $$\begin{array}{c} F_{D}=-\nabla U_{\gamma } \end{array}$$

The surface energy gradient of a solid surface is typically achieved by constructing a wettability gradient, which can be realized in two common ways: by creating variations in the surface’s microstructure or by altering its chemical composition. These are referred to as roughness-induced wettability gradients and chemical wettability gradients, respectively [86]. By introducing a roughness gradient on a solid surface, an apparent roughness-induced wettability gradient can be formed. Due to differences in the distribution of surface roughness, regions with denser microstructures can significantly enhance the hydrophilicity (for intrinsically hydrophilic materials) or hydrophobicity (for intrinsically hydrophobic materials) of the surface, thereby creating an apparent roughness-induced wettability gradient that drives droplet motion.

The surface energy (*γ_sg_*) of hydrophilic regions is always higher than that of hydrophobic regions, and droplets tend to migrate from areas of lower surface energy to those of higher surface energy. Therefore, constructing a surface that combines hydrophilic and hydrophobic regions on the same substrate generates a surface energy gradient, which drives droplet motion. When the intermolecular interactions at different locations on the surface vary, the surface energy gradient induces a solid–liquid interfacial tension gradient, leading to variations in *γ*_sl_ at different positions. Typically, a chemical wettability gradient is determined by the chemical functional groups present on the solid surface, and the driving force of this chemical wettability gradient can be expressed as:

(14) $$F_{chem}=\gamma (\cos\theta_{ad}-\cos\theta_{re})$$

By modifying the chemical functional groups on the solid surface, *γ_sg_* can be altered, thereby affecting the intrinsic contact angle of the droplet. This is also a common method for adjusting the apparent contact angle of droplets.

#### 2.3.2. Laplace Pressure Gradient

The Young–Laplace equation describes the relationship between the additional pressure across a curved liquid surface, the liquid’s surface tension, and the radii of curvature. In brief, it can be used to determine the shape of a liquid surface; however, analytical solutions exist only for a limited number of cases. Therefore, in most situations, the Young–Laplace equation must be solved numerically. The additional pressure difference across any surface can be expressed as:

(15) $$\Delta P=\gamma \left(\frac{1}{r_{1}}+\frac{1}{r_{2}}\right)$$

where Δ*P* denotes the pressure difference between the inside and outside of the liquid surface, *γ* is the surface tension coefficient, and *r*_1_ and *r*_2_ are the principal radii of curvature of the surface. The surface tension of a curved liquid surface subjects it to an additional pressure, which always acts toward the center of curvature and is related to the curvature value.

When a droplet is on a conical structure (Figure 5b), the asymmetry of the cone induces an imbalance in surface tension, creating a pressure difference between the inside and outside of the liquid surface—this is the Laplace pressure difference, Δ*P_Laplace_*. When the droplet wets the outer surface of the cone, it experiences a pressure driving it from the end with a smaller radius of curvature toward the end with a larger radius of curvature. The magnitude of this pressure is given by [87]:

(16) $$\begin{array}{c} \Delta P_{Laplace}=-\int_{r_{2}}^{r_{1}}\frac{2\gamma }{{\left(R+R_{0}\right)}^{2}}\sin\alpha dx \end{array}$$

where Δ*P_Laplace_* is the Laplace pressure difference, *R* is the local radius of the droplet’s cross-section on the cone surface, *r*_1_ and *r*_2_ are the local radii on either side of the droplet’s cross-section, *R*_0_ is the droplet radius on the cone, *α* is half the cone’s tip angle, and *dx* is the integration variable for a single cone.

When the droplet is inside a conical structure (Figure 5c), the Laplace driving force generated by the asymmetry is:

(17) $$\begin{array}{c} \Delta P_{Laplace}=\frac{4\gamma \cos\beta }{x_{B}\alpha }-\frac{4\gamma \cos\beta }{x_{A}\alpha } \end{array}$$

where *β* is the contact angle between the droplet and the inner surface. In this case, the direction of the Laplace pressure depends on the surface wettability (contact angle *β*). When *β* < 90°, as shown in Figure 5c, the inner surface is hydrophilic, the solid–liquid interface forms a concave meniscus, and the droplet moves toward the cone’s tip. When *β* > 90°, the inner surface is hydrophobic, the interface forms a convex meniscus, and the droplet moves toward the cone’s base [87].

Both the Laplace pressure gradient and the wettability gradient can drive directional droplet motion, and in some cases, these two forces act synergistically to produce a more effective driving effect.

## 3. Biomimetic Fog Water Collection Structures

### 3.1. Namib Desert Beetle

In the southwestern region of Africa lies an ancient and arid desert known as the Namib Desert. With an average annual rainfall of only 2–200 mm and an average annual temperature ranging from 9 to 20 °C, the area remains in a state of persistent aridity [88]. Nevertheless, a variety of life forms inhabit this harsh environment, where the dry climate has driven local flora and fauna to evolve unique physiological traits and behaviors [89,90,91]. The Namib Desert beetle is one such species, capable of meeting its water requirements by harvesting fog droplets in the early morning [92].

#### 3.1.1. Fog Water Collection Mechanism of the Namib Desert Beetle

**Figure 6:**
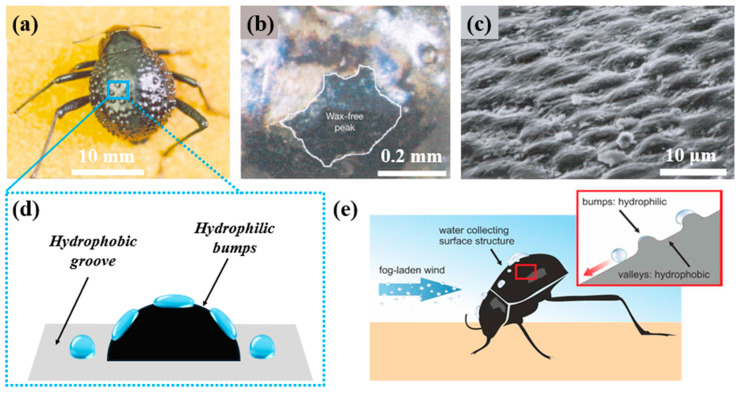
Fog Water Collection Mechanism of the Namib Desert Beetle. (**a**) Dorsal structure of the Namib Desert beetle. (**b**) A hydrophilic protrusion on the dorsal surface. (**c**) Scanning electron micrograph of the hydrophobic groove region (Reprinted with permission from ref. [38], Copyright © 2001, Springer Nature Limited). (**d**) Schematic illustration of a droplet adsorbed by a dorsal hydrophilic protrusion; (**e**) Schematic of fog water collection by the Namib Desert beetle (Reprinted with permission from ref. [98], Copyright ©2018, American Chemical Society).

In 2001, Park et al. [38] discovered that the Namib Desert beetle collects early-morning fog in the desert through a cooperative mechanism involving hydrophilic protrusions and hydrophobic grooves on its back. A single beetle can harvest fog water equivalent to up to 12% of its body weight in one collection event. Although the quantity and efficiency of fog water collected by the beetle are lower than those of trees, this mechanism reveals the secret of surface wettability [93]. As shown in Figure 6a, the beetle’s back is covered with numerous irregularly arranged protrusions, spaced approximately 0.5–1.5 μm apart. Each protrusion has a smooth surface (Figure 6b) and exhibits a certain degree of hydrophilicity. The grooves between adjacent protrusions are composed of hydrophobic waxy material, forming hemispherical structures arranged in a regular hexagonal pattern at the microscale, with each hemisphere having a diameter of about 10 μm (Figure 6c). This hydrophobicity is similar to that of a lotus leaf surface [94,95]. As illustrated in Figure 6d, when fog flows arrive in the desert, the beetles tilt their bodies toward the fog flow and raise their backs to better intercept it. Microdroplets in the fog are adsorbed upon contacting the hydrophilic protrusions, whereas those striking the hydrophobic grooves may rebound toward the hydrophilic protrusions, where they are re-adsorbed or cause the droplets already present to grow rapidly [96]. Once the droplets enlarge sufficiently to cover the entire hydrophilic protrusion, gravity causes them to detach and travel along the hydrophobic grooves toward the beetle’s mouth, meeting its water needs [97]. The alternating hydrophilic/hydrophobic pattern on the beetle’s back plays a crucial role in fog water collection, enhancing fog water capture efficiency while enabling rapid droplet removal [98].

#### 3.1.2. Fog Water Collection Structures (FWCSs) and Fabrication Inspired by the Namib Desert Beetle

**Figure 7:**
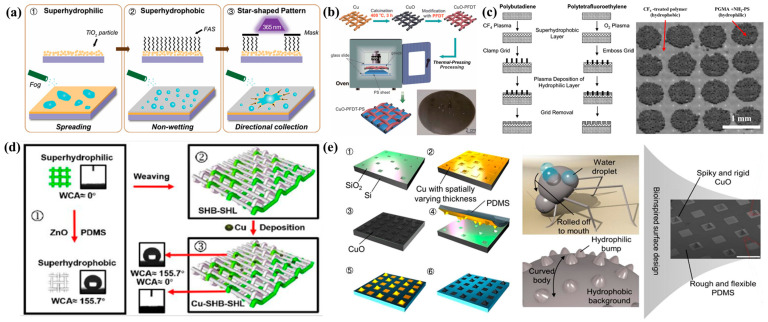
FWCSs inspired by the Namib Desert beetle. (**a**) Schematic illustration of a surface with star-shaped hydrophilic–hydrophobic patterns (Reprinted with permission from ref. [100], Copyright ©2014 WILEY-VCH Verlag GmbH & Co. KGaA, Weinheim). (**b**) Schematic of a hydrophobic-modified metal mesh attached to a hydrophilic substrate [101]. (**c**) Hydrophilic–hydrophobic surface prepared by two-step plasma deposition (Reprinted with permission from ref. [102], Copyright ©2007, American Chemical Society). (**d**) Schematic of Cu-SHB-SHL fabric fabrication (Reprinted with permission from ref. [104], Copyright ©2021, Elsevier). (**e**) A flexible hydrophilic-hydrophobic composite 3D surface inspired by the water collection mechanism on the back of the Namib Desert beetle [105]. The main method involves depositing Cu on a Si substrate through two steps of electroplating and then oxidizing it to complete the preparation.

In general, FWCSs inspired by the Namib Desert beetle require at least two distinct wettability—hydrophilic and hydrophobic—to create a wettability gradient that enables fog water collection. The greater the wettability gradient, the more pronounced the advantage of the FWCS in fog water collection [99]. Bai et al. [100] designed and fabricated a surface with a star-shaped pattern exhibiting different wettability. As shown in Figure 7a, hydrophilic titanium dioxide (TiO_2_) slurry was deposited as a film on a bare glass substrate via spin coating, followed by treatment with hydrophobic reagent to convert the surface from superhydrophilic to superhydrophobic. Using a star-shaped patterned mask, ultraviolet light was selectively irradiated onto the exposed FAS-coated film, inducing photocatalytic decomposition and restoring the TiO_2_ surface to a superhydrophilic state. The resulting star-patterned hydrophilic–hydrophobic surface utilized both the surface energy gradient and the Laplace pressure gradient to rapidly drive small water droplets toward the more hydrophilic regions. Therefore, directly employing chemical methods to fabricate surfaces with different wettability can yield improved fog water collection performance. Although patterned masks allow faster and more precise fabrication of hydrophilic–hydrophobic surfaces, diffraction effects inevitably reduce pattern accuracy and clarity. Wang et al. [101] employed a hot-pressing method to attach a superhydrophobic CuO–PFDT-modified metal mesh onto a hydrophilic polystyrene (PS) substrate, thereby producing a hydrophilic–superhydrophobic composite surface (Figure 7b) to enhance fog water efficiency. This structure allows control of the cavity dimensions in the hydrophilic regions by adjusting the hot-pressing temperature, thereby altering the mesh number to achieve an optimized FWCS design. In contrast to the above two approaches, which fabricate hydrophobic/superhydrophobic regions on hydrophilic surfaces, R. P. Garrod et al. [102] created hydrophilic spots on a superhydrophobic substrate via pulsed plasma deposition, producing a hydrophilic–hydrophobic patterned surface (Figure 7c). The superhydrophobic substrate was prepared by CF_4_ plasma fluorination of polybutadiene and oxygen plasma etching of polytetrafluoroethylene (PTFE). Each superhydrophilic spot measured 500 µm in diameter, with a spacing of 1000 µm between adjacent spots. This design mimicked the dimensional parameters of the beetle’s dorsal surface, and the superhydrophilic spots (4-vinylpyridine) achieved the highest fog water efficiency. With the development of flexible materials, textiles and fabrics have increasingly been used for fog water collection. Xin et al. [103] modified the hydrophilic surface of sponge fabric using a temperature-responsive polymer via a “grafting” method, producing a multifunctional and simple sponge fabric. This fabric can autonomously collect and release water from humid air in response to diurnal temperature variations in desert environments. The combination of the temperature-responsive polymer with the highly rough fabric surface enables reversible switching between superhydrophobic and superhydrophilic states over multiple cycles. As shown in Figure 7d, Lai et al. [104] wove superhydrophilic and superhydrophobic polyester yarns into a fabric and deposited Cu particles onto its surface, producing a hydrophilic–hydrophobic composite fabric. Compared with conventional planar structures, this fabric exhibited a higher fog water efficiency of 1432.7 mg·h^−1^·cm^−2^, primarily due to the enhanced fog condensation from the Cu particle deposition and the synergistic effect between yarns of different wettability. This approach enables low-cost, high-efficiency fog water collection. Park et al. [105] fabricated a flexible composite 3D surface consisting of hydrophilic CuO protrusions on a rough hydrophobic polydimethylsiloxane (PDMS) substrate (Figure 7e). The combination of a wettability gradient with a periodically arranged 3D curved surface structure resulted in significantly higher fog water efficiency, even under suboptimal fog-flow conditions. The fog water collection rate of this 3D surface was 16 times greater than that of a planar 2D hybrid surface. Their study demonstrated that 3D Namib Desert beetle-inspired structures offer superior performance in fog water collection.

Based on the above studies, efficient biomimetic FWCSs inspired by the Namib Desert beetle should satisfy two requirements: (i) one component should preferably be a flexible textile, and (ii) the other component should exhibit surface wettability opposite to that of the textile, so that together they form a structure with a wettability gradient, thereby enabling efficient fog water collection.

### 3.2. Cactus Spines

In deserts, in addition to the Namib Desert beetle’s ability to perform fog water collection, cacti can also survive due to their unique spine structures [106]. Consequently, cacti have become one of the favored models for scientists when designing fog water collection structures [107].

#### 3.2.1. Fog Water Collection Mechanism of Cactus Spines

**Figure 8:**
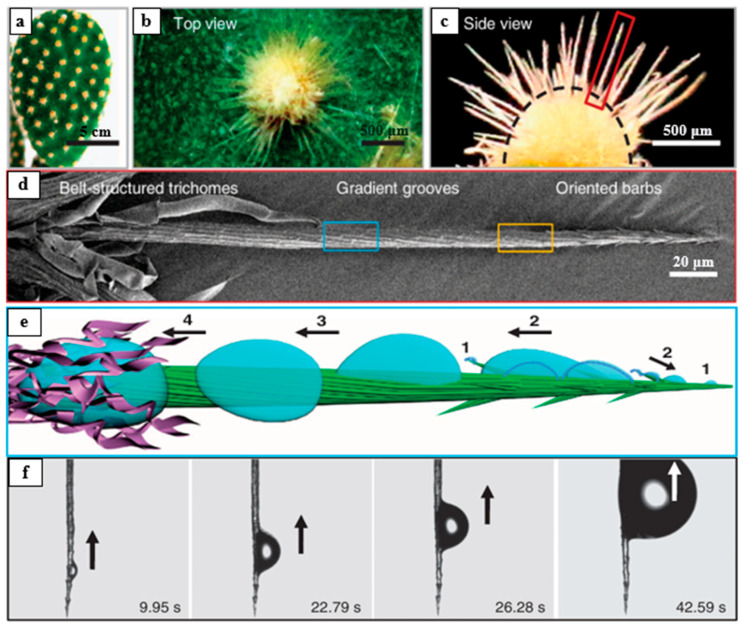
Fog Water Collection Mechanism of Cactus Spines. (**a**) Optical image of a cactus. (**b**,**c**) Magnified optical images showing clusters of spines and trichomes distributed on the stem; (**d**) SEM image of a single spine. (**e**) Process of fog water collection, in which water droplets undergo deposition (1), collection (2), and transportation (3), and are rapidly absorbed into the cactus stem upon contact with the trichomes (4). (**f**) Directional movement of a droplet along a cactus spine positioned at –90° (Reprinted with permission from ref. [109], Copyright ©2012, Springer Nature).

It is well known that plants of the Cactaceae family are renowned for their extreme drought tolerance (Figure 8a). Researchers have investigated the structures and fog water collection capabilities of various cactus species [39]. As shown in Figure 8b,c, these plants have evolved unique structural features over long periods of adaptation: clusters of spines and trichomes are distributed on their thick, succulent stems [108]. Observations of individual spines reveal that most are hydrophilic and consist of three distinct structural regions, each playing a different role in fog water collection. The tip of the spine is covered with directionally arranged conical barbs; the middle section features inclined surfaces and grooves of varying roughness; and the base is covered with belt-like trichomes (Figure 8d) [109]. From the perspective of the tip morphology, cactus spines exhibit either a conical or triangular cross-sectional shape. These specific geometric dimensions induce a Laplace pressure gradient, which facilitates the directional movement of water droplets from the tip toward the base [110]. The integration of these multiscale surface structures induces the combined action of both driving forces, enabling droplets attached to the spine to be rapidly and directionally transported from the conical tip to the base, thereby enhancing the cactus’s fog water collection efficiency.

As shown in Figure 8e, the fog water collection process of cactus spines begins with the initial deposition of microdroplets on the main spine and its barbs [109], followed by their directional movement along these structures. As deposition continues and microdroplets coalesce, their size increases until they detach from the tip of the main spine. The larger droplets are then further transported along grooves with a surface energy gradient and absorbed by the hydrophilic trichomes at the base of the main spine. This sequence—fog water capture, convergence, transport, and absorption—occurs repeatedly on the cactus spine, forming a continuous fog water collection mechanism. The collected droplets do not fall to the ground but are instead directly absorbed into the cactus interior. As shown in Figure 8f, even when the cactus spine is positioned at −90°, the combined effects of the surface energy gradient and the Laplace pressure gradient can overcome gravity to transport droplets from the tip to the base of the spine.

#### 3.2.2. FWCSs and Fabrication Inspired by Cactus Spines

##### Fabrication of a Single Conical FWCSs Inspired by Cactus Spines

**Figure 9:**
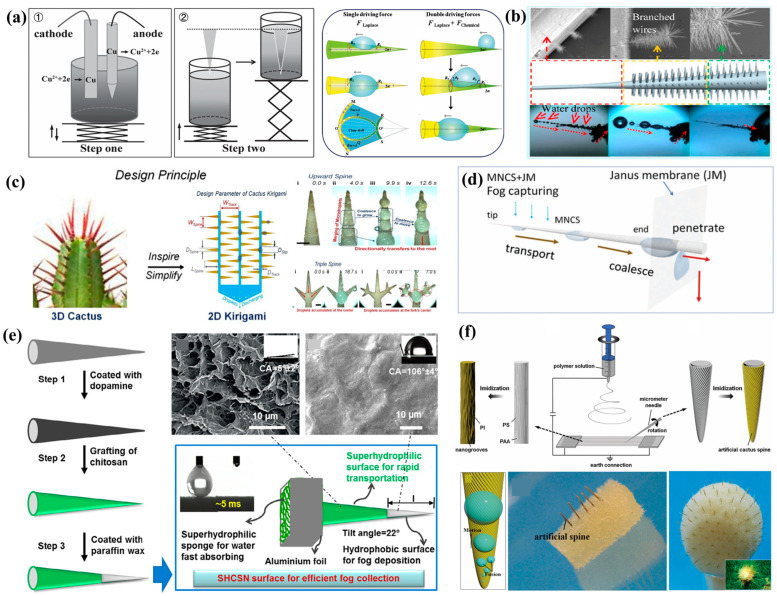
Fabrication of a Single Conical FWCSs Inspired by Cactus Spines. (**a**) Schematic illustration of a cactus-spine-inspired structure with a wettability gradient prepared by a two-step electrochemical etching method (Reprinted with permission from ref. [108], Copyright ©2013 WILEY-VCH Verlag GmbH & Co. KGaA, Weinheim). (**b**) Schematic illustration of a fog water collection structure composed of ZnO nanowires grown on ZnO rods, the microscopic schematic diagram of ZnO nanowires and the image of water collection by ZnO rods (Reprinted with permission from ref. [111], Copyright ©2014, American Chemical Society). (**c**) Schematic illustration of a fog water collection structure derived from papercutting patterns simplified from 3D to 2D and a 3D diagram of the fog water collection system (Reprinted with permission from ref. [112], Copyright ©2020, Royal Society of chemistry). (**d**) Schematic illustration of a fog water collection system integrating conical micro/nanostructures with a Janus membrane (Reprinted with permission from ref. [113], Copyright © 2018 WILEY-VCH Verlag GmbH& Co. KGaA, Weinheim). (**e**) Schematic illustration of a fog water collection system combining conical structures with wettability gradients and a sponge (Reprinted with permission from ref. [114], Copyright ©2018 Elsevier Inc. All rights reserved.). (**f**) Schematic illustration of an artificial cactus-spine structure fabricated by electrospinning and the fog water collection process (Reprinted with permission from ref. [115], Copyright ©2015 The Authors. Published by WILEY-VCH Verlag GmbH & Co. KGaA, Weinheim).

Inspired by the fog water collection mechanism of individual cactus spines, Ju et al. [108] fabricated a single conical structure with a wettability gradient, as shown in Figure 9a, using a two-step electrochemical etching method. This conical structure possessed both hydrophilic and hydrophobic regions. Force analysis revealed that the combination of chemical wettability gradient force and Laplace pressure altered the droplet shape on the cone surface, and the dual-gradient driving forces accelerated the directional transport of droplets toward the base, achieving a faster transport rate than that of a cone driven solely by Laplace pressure. Heng et al. [111] grew conical ZnO nanowire branches on a conical ZnO rod to create a single cactus-spine-inspired FWCSs with a total length of approximately 3.4 mm (Figure 9b). Droplets deposited on the ZnO rod were transported to the base of the conical nanowires under the combined action of Laplace pressure and capillary force, where they were ultimately collected. This ZnO rod was capable of collecting approximately 6 μL of water within 30 min—1.5 times the fog water collection efficiency of a uniform smooth cone—demonstrating that the barb-like structures on cactus spines enhance fog water collection performance. Bai et al. [112], inspired by cactus spines, designed a foldable paper-cut pattern in which the 3D conical spine structure of the cactus was simplified into a 2D triangular paper cut. As shown in Figure 9c, the wax-infused triangular paper cut can replicate the fog water collection function of cactus spines, effectively capturing fog droplets and rapidly refreshing the surface through directional droplet transport, thereby enabling continuous collection. Fluid simulations indicated that spines with smaller apex angles can provide higher fog-flow velocities, thereby improving fog water collection performance. Under a fog flow of approximately 220 cm/s, the cactus paper cut achieved a water collection rate of about 4000 mg·cm^−2^·h^−1^, which is 1.6 times and 11 times greater than those of harp-shaped and plate-shaped FWCSs, respectively. Zhou et al. [113] proposed a fog water collection system integrating conical spines with micro/nanostructures and a Janus membrane (MNCS + JM) (Figure 9d). In this system, the conical spines, whose surfaces are covered with rough micro/nanostructures (MNCS), can rapidly capture tiny fog droplets and transport them to the base, while the Janus membrane with a wettability gradient collects the droplets directionally transported along the spine surface. Chen et al. [114] coated both hydrophilic and hydrophobic paints onto the same cone to fabricate a single conical structure with a wettability gradient. A sponge was attached to the base of the cone to rapidly absorb directionally transported droplets, thereby facilitating the continuous fog water collection cycle (Figure 9e). Experimental analysis showed that the front section of the cone, serving as the hydrophobic region, had an optimal length of 2.6 mm, enabling rapid deposition of microdroplets from the fog. The rear section was superhydrophilic, where a gradually formed liquid film allowed droplets to be quickly transported to the base and absorbed by the sponge. The fog water collection efficiency was approximately 2.3 times and 4.2 times higher than that of uniformly hydrophobic and uniformly hydrophilic conical surfaces, respectively.

To realistically replicate the surface roughness of cactus spines, Bai et al. [115] fabricated artificial cactus spines with well-defined grooved structures by combining electrospinning with the sacrificial template method. A total of 180 fabricated artificial spines were assembled onto a spherical sponge to create an artificial cactus model (Figure 9f). This method enables precise construction of microgrooved structures mimicking cactus spine surfaces; however, it suffers from low production yield and high energy consumption.

##### Fabrication of Conical Array FWCSs Inspired by Cactus Spines

**Figure 10:**
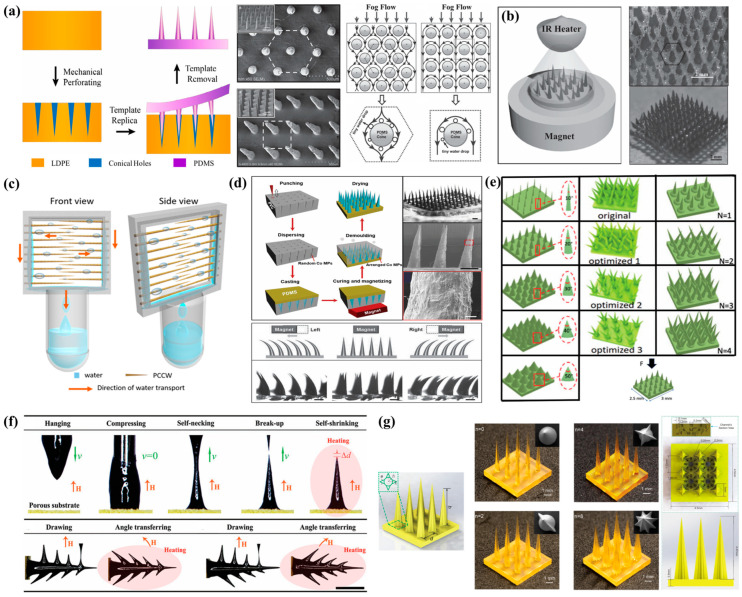
Fabrication of conical array FWCSs inspired by cactus spines. (**a**) Schematic illustration of PDMS conical arrays arranged in hexagonal and quadrilateral patterns and the influence of square arrangement and hexagonal arrangement on the fog flow (Reprinted with permission from ref. [116], Copyright ©2014 WILEY-VCH Verlag GmbH & Co. KGaA, Weinheim). (**b**) Schematic illustration of conical needle tips fabricated by a magnetic-particle-assisted molding method (Reprinted with permission from ref. [117], Copyright ©2014 WILEY-VCH Verlag GmbH& Co. KGaA, Weinheim). (**c**) Schematic illustration of a fog water collection structure based on an array of periodically roughness-gradient conical copper wires (PCCWs) (Reprinted with permission from ref. [118], Copyright ©2016, American Chemical Society). (**d**) Schematic illustration of conical arrays fabricated by a magnetic-particle-assisted molding method and by using magnets to control the swinging of the cones to collect fog water (Reprinted with permission from ref. [119], Copyright ©2015 WILEY-VCH Verlag GmbH & Co. KGaA, Weinheim). (**e**) Schematic illustration of a multi-branched conical array surface fabricated by 3D printing and the effects of different quantities, arrangements, and tip angle parameters (Reprinted with permission from ref. [120], Copyright ©2019 WILEY-VCH Verlag GmbH & Co. KGaA, Weinheim). (**f**) Schematic flow chart of conical structures with multidirectional microbarbs fabricated on a superhydrophilic porous substrate via magnetorheological drawing lithography (Reprinted with permission from ref. [121], Copyright ©2019 WILEY-VCH Verlag GmbH & Co. KGaA, Weinheim). (**g**) Schematic illustration of a hybrid fog water collection structure fabricated by 3D printing (Reprinted with permission from ref. [122], Copyright ©2021, American Chemical Society).

To further improve the fog water collection efficiency of cactus-spine-inspired structures, the most direct approach is to increase the number of biomimetic spines. Since the template method allows large-scale replication of cactus spine structures, Ju et al. [116] combined mechanical perforation with the template method to fabricate PDMS conical array surfaces with different arrangement patterns. As shown in Figure 10a, the hexagonally arranged conical array surface exhibited a clear advantage over the quadrangular arrangement. Studies revealed that the hexagonal conical array enhanced the transport efficiency of fog flow and accelerated droplet deposition. Moreover, the deposited droplets moved rapidly and directionally along each cone, refreshing the water collection cycle of the conical array surface. This rapid regeneration of droplets on the cone surfaces further enhanced the fog water collection efficiency. Cao et al. [117] employed a magnetic-field-induced, magnetic-particle-assisted molding approach to fabricate cactus-spine-like conical microneedle arrays from PDMS and magnetic particles (MPs) using a modified magnetic particle-assisted molding (MPAM) method (Figure 10b). By adjusting the ratio of PDMS to MPs, the structural morphology of the microneedle tips could be precisely controlled. Integrating the hydrophobic conical microneedle array with a hydrophilic sponge substrate resulted in a novel artificial cactus fog water collection structure capable of spontaneously and continuously collecting, transporting, and storing droplets obtained from fog water collection. Xu et al. [118] fabricated an artificial conical copper wire with periodic variations in wettability and surface roughness, inspired by the structures of cactus spines and spider silk. Fog water was captured at periodically arranged sites along the conical wire surface. Driven by the Laplace pressure difference generated from the geometric gradient of the cone and the surface wettability gradient arising from periodic changes in surface roughness, the coalesced droplets were transported toward the base of the wire (Figure 10c). Their study further demonstrated that the tilt angle of the conical wire influences its fog water collection capability. Moreover, the fog water collection efficiency depends not only on the surface properties of the collection material but also on the ambient wind speed.

In general, FWCSs perform better in fog flows with a certain velocity, exhibiting passive collection behavior. In some regions, however, low fog flow speeds lead to reduced fog water collection efficiency. Inspired by cactus spines and magnetic responsiveness, Peng et al. [119] developed a device suitable for fog water collection under static atmospheric conditions. They fabricated cactus-spine-mimicking conical arrays by combining mechanical perforation with template dissolution. As illustrated in Figure 10d, a permanent magnet was placed beneath the sample, causing the magnetic Co-MPs embedded in PDMS to align under the magnetic field and preferentially deposit at the cone tips. After demolding, conical arrays resembling cactus spines were obtained. Under the influence of the magnetic field, the cone tips oscillated in the direction of the field, effectively addressing the limitation of fixed-orientation conical arrays in capturing fog from multiple directions, thereby enhancing fog water collection efficiency. Although the template method enables rapid, large-area fabrication of cactus-spine-inspired conical arrays, it has drawbacks: the fabricated cones are limited in length, and the structures are prone to breaking inside the mold during demolding. Moreover, the barbs on natural cactus spines play an indispensable role in water collection, yet the template method cannot accurately replicate these fine structures.

With the advancement of technology, 3D printing has been widely employed to fabricate complex, high-precision micro/nanostructures [123,124,125,126]. Researchers have attempted to use high-resolution 3D printing equipment to produce intricate cactus-spine-inspired structures or conical arrays, with photosensitive resin being a commonly used biomimetic printing material. Mahmood et al. [127] utilized 3D printing technology to fabricate various types of conical arrays and conducted an in-depth analysis of their fog water collection mechanisms. They investigated the effects of structural parameters of individual cones, such as cone tip angle (*θ*), cone height (*H*), and spacing between cones (*S*) on fog water collection efficiency. In addition, they examined the influence of operational parameters, including the distance (*d*) between the humidifier and the conical array, the inclination angle (*α*) relative to the fog flow, and surface wettability, on fog water collection efficiency. The results indicated that the highest fog water collection efficiency was achieved when the cones were oriented parallel to and positioned close to the fog flow. Li et al. [120] fabricated a multi-branched conical array surface using 3D printing technology and sputtered a nanoscale hydrophobic coating onto the array surface to accelerate water growth. As shown in Figure 10e, they investigated the effects of several factors on fog water collection efficiency, including the cone tip angle (*θ*) of individual cones in the array, the number of cones at a single site, the fog flow direction, cone strength, and cone arrangement pattern. The results indicated that a hexagonally arranged conical array surface enhanced the surrounding fog flow, and when the cone tip angle (*θ*) was 10°, the fog water collection mass increased by 2 mg·min^−1^·mm^−3^. This study offers an intriguing prospect for designing spatially specialized structures with biomimetic features to achieve energy-free and highly efficient fog water collection. Yi et al. [121] employed a magnetorheological drawing lithography (MRDL) method to fabricate conical structures with microbarbs oriented in different directions on a superhydrophilic porous substrate. As shown in Figure 10f, under an external magnetic field, the formation of microbarbs on the conical structures can be divided into two main stages: microbarb stretching and microbarb angle adjustment. In a vertical magnetic field, rows of microbarbs perpendicular to the cone surface can be stretched out. The angle between the microbarbs and the conical structure can be tuned by adjusting the direction of the external magnetic field. Notably, conical structures with backward-oriented microbarbs significantly accelerated the directional transport of water droplets, achieving higher fog water collection efficiency. These properties are primarily attributed to the combined effects of the Laplace pressure gradient generated by the conical shape and microbarbs, the capillary pressure arising from the concave meniscus between the cone and the backward barbs, and the wettability of the superhydrophilic porous substrate. Furthermore, the team assembled an annular fog water collection device by integrating the conical structures with a sponge. In this system, droplets captured by the cones flowed toward the base and were ultimately absorbed by the sponge. The timely transport of droplets along the conical structures enabled continuous fog water capture and high-efficiency fog water collection. Liu et al. [122] fabricated a bioinspired hybrid FWCS via 3D printing, consisting of cactus-spine-mimicking structures with longitudinal surface ridges and bottom channels decorated with curved inclined arc-pitted grooves (C-IAPGs) (Figure 10g). Experimental results showed that cactus-spine structures with four longitudinal ridges achieved the highest fog water collection efficiency, while the incorporation of C-IAPG bottom channels enabled rapid and efficient transport of collected droplets into the bottom reservoir. This work provides valuable inspiration for the development of next-generation hybrid FWCSs.

Inspired by the fog water collection capability of cactus spines, various fabrication methods have been employed to produce conical structures with diverse geometries and fractal features. The fog water collection and directional droplet transport efficiency of such conical structures depend on the Laplace pressure difference generated by the cone tip angle, the surface roughness of the cone, and the surface wettability gradient. Studies have shown that, for a fixed cone height, an optimal tip angle is approximately 10°. Moreover, a design in which the cone tip is hydrophobic and the base is hydrophilic is more favorable for fog droplet deposition, capture, and directional transport. In addition, the incorporation of barb-like features and clustered arrangements of multiple cones at a single site can, to some extent, enhance fog water collection efficiency. However, the durability of these conical structures remains to be fully evaluated, and their fabrication processes are often complex, with high production difficulty, which limits their applicability. Further research and optimization of cactus-spine-inspired fabrication techniques are needed to improve durability and broaden applicability. Exploring a wider range of biomimetic structures and integrating them with cactus-spine-inspired designs may better address diverse environmental conditions and operational requirements.

### 3.3. Spider Silk

Spider webs are regarded as hunting grounds for spiders, and the silk fibers that compose them have been extensively studied due to their outstanding properties, including high toughness, high tensile strength, and excellent elasticity [128]. The diameters of spider silk fibers range from micrometers to millimeters, and the differences between various types of spider silk are difficult to distinguish with the naked eye [129]. In addition, spider silk exhibits a remarkable sensitivity to water. In nature, there is a great diversity of spider species, and some produce silk with specialized fog water collection capabilities. The unique structural features of spider silk have also served as a source of inspiration for scientists in the development of fog water collection material [130,131].

#### 3.3.1. Fog Water Collection Mechanism of Spider Silk

**Figure 11:**
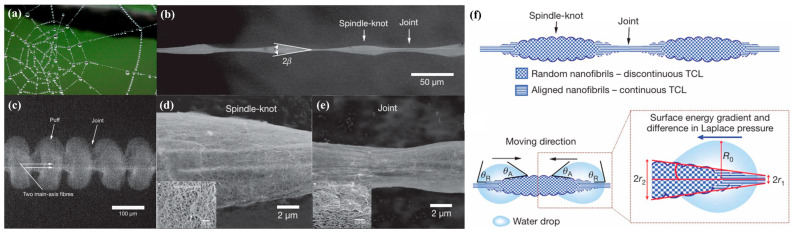
Fog water collection mechanism of spider silk. (**a**) Photograph showing droplets collected on spider silk in the early morning. (**b**) SEM image of the periodic spindle-knot structure of spider silk. (**c**) SEM image of the beaded structure composed of numerous nanofibers under dry conditions. (**d**,**e**) SEM images of the spider silk structure in the “wet-reconstructed” state, showing periodic spindle-knots (**d**) and slender joints (**e**). (**f**) Schematic illustration of the principle of droplet movement on spider silk (Reprinted with permission from ref. [40], Copyright ©2010, Macmillan Publishers Limited. All rights reserved).

As shown in Figure 11a, on early mornings in the wild, spider silk in humid air is often seen covered with numerous water droplets. In 2010, Zheng et al. [40] discovered that the ability of *Uloborus walckenaerius* spider silk to capture fog and collect water arises from its unique periodic spindle-knot structure. Moreover, the morphology of the silk differs between dry conditions and wetted conditions after fog exposure. The researchers fabricated periodic spindle-knot structures by thermally drawing nylon fibers and conducted fog water collection experiments to investigate their performance.

The fog water collection capability of spider silk is attributed to its unique fiber structure, which consists of periodic spindle-knots and joints [40], as shown in Figure 11b. Spider silk exhibits two distinct morphologies under dry and wet conditions (Figure 11b–e). When dry silk is exposed to fog (Figure 11c), its structure changes as fog water condenses, entering a “wet-rebuilt” state in which periodic spindle-knots are formed. In this state, the spindle-knots are composed of densely interwoven random nanofibrils (Figure 11d), whereas the joints consist of relatively well-aligned nanofibrils (Figure 11e). The spindle-knots possess greater surface roughness than the joints, and the anisotropy of their surface structure generates a surface energy gradient, giving the spindle-knots higher surface energy than the joints. In addition, due to the conical geometry of the spindle-knots (Figure 11b, tip angle = 2*β*), a Laplace pressure difference is established between the high-curvature regions (joints) and the low-curvature regions (spindle-knots). The synergistic action of these two factors drives droplets collected from fog water to move from the joints toward the spindle-knots (Figure 11f) [40]. While either a surface energy gradient or a Laplace pressure difference alone can drive submillimeter-sized droplets, the pronounced contact angle hysteresis of micrometer-sized droplets makes it difficult for either force alone to induce directional motion. Spider silk achieves this by harnessing the combined effect of both driving forces.

The discovery of the fog water collection mechanism of spider silk has brought about a revolutionary breakthrough in the field of fog water-harvesting. As a result, the focus of biomimetic fog water collection research has shifted from gravity-driven collection to the design of FWCSs and the wettability of their surfaces, establishing the central role of gradient-interface materials in fog water collection.

#### 3.3.2. Fabrication of FWCSs Inspired by Spider Silk

**Figure 12:**
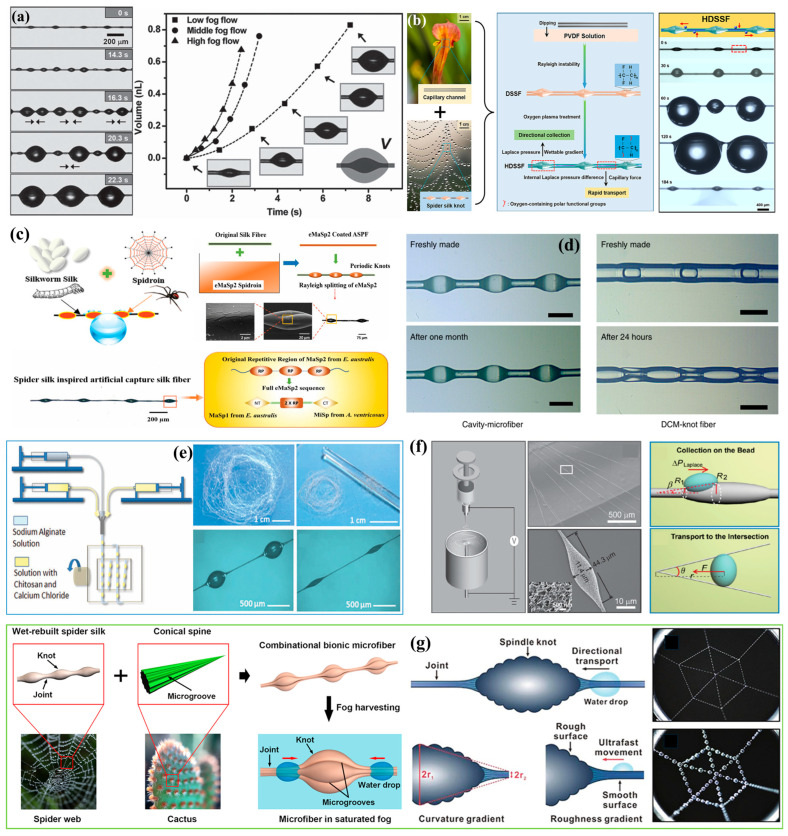
Fabrication of FWCSs inspired by spider silk. (**a**) Photograph and parametric study of fog water collection on spindle-knot structures fabricated by dip-coating (Reprinted with permission from ref. [137], Copyright © 2011 WILEY-VCH Verlag GmbH & Co. KGaA, Weinheim). (**b**) Schematic illustration of the biomimetic design and fabrication of dual-thread spider silk fibers, along with photographs of fog water collection over time (Reprinted with permission from ref. [139], Copyright © 2023 Elsevier B.V All rights reserved). (**c**) Schematic illustration of spider-capture-silk fabricated using recombinant techniques (Reprinted with permission from ref. [142], Copyright © 2020 WILEY-VCH Verlag GmbH & Co. KGaA, Weinheim). (**d**) Photograph of fog water collection on bioinspired cavity microfibers (Reprinted with permission from ref. [144], Copyright © 2017, Springer Nature). (**e**) Schematic illustration of spider silk-inspired microfibers fabricated by microfluidics, with photographs of the fibers before and after wet reconstruction (Reprinted with permission from ref. [145], Copyright © 2019 WILEY-VCH Verlag GmbH & Co. KGaA, Weinheim). (**f**) Schematic illustration of bioinspired multiscale fog water collection membranes fabricated by electrospinning, together with a schematic diagram of droplet force analysis on the structure (Reprinted with permission from ref. [146], Copyright © 2016 WILEY-VCH Verlag GmbH& Co. KGaA, Weinheim). (**g**) Schematic illustration of spindle-knot fibers with grooved textures fabricated by dynamic interfacial spinning (DIS), and photographs of their fog water collection processes (Reprinted with permission from ref. [147], Copyright © 2022 Elsevier B.V All rights reserved).

Because spider-silk-inspired structures are similar to the fog nets that have already been applied on a large scale, research on spider-silk-inspired fog water collection structures and materials has developed rapidly in recent years [132,133,134,135]. The spindle-knot structure of spider silk determines its fog water collection performance; therefore, the key to fabricating spider-silk-inspired fog water collection structures lies in the preparation of spindle-knot structures [136]. Bai et al. [137] fabricated artificial spindle-knot structures by immersing uniform nylon fibers into a poly (methyl methacrylate) (PMMA) solution and then drawing them out horizontally. Under the action of Rayleigh instability, the PMMA solution coating on the nylon fibers contracted into spindle-knot structures resembling those of natural spider silk. The fabricated artificial spindle-knot structures were applied to fog water collection and microdroplet manipulation. Experimental results indicated that the fabrication environment and the dimensional morphology of the artificial spindle-knots had a significant influence on droplet manipulation (Figure 12a). Subsequently, uniform nylon fibers were immersed in PMMA solutions of different concentrations, and by varying the horizontal withdrawal speed, PMMA spindle-knots with controllable dimensions and morphologies were obtained. The optimized artificial spindle-knot structures achieved a maximum water collection performance of 10.44 μL·h^−1^ per spindle-knot, capturing more water from fog than uniform nylon fibers without PMMA coating. Furthermore, Chen et al. [138] prepared a nylon mesh with graded structures (NMGS) by spraying poly (methyl methacrylate) (PMMA) onto the nylon mesh and depositing PMMA at the intersections. At a relative fog flow rate of 110 g·s^−1^·m^−2^, the NMGS achieved the lowest load mass and a fog water collection efficiency of 1688 mg·cm^−2^·h^−1^. Although conventional spider-silk-inspired fibers exhibit high fog water collection capability, they perform less effectively in the rapid transport of deposited droplets, which may hinder further improvement in fog water collection efficiency. In fact, the main axis of natural spider silk consists of two independent parallel lines, a structural feature that may be an important factor contributing to its high fog water collection efficiency [40]. Huan et al. [139], inspired by spider silk and the *Nepenthes mirabilis*, fabricated a novel hydrophilic dual-thread spider silk fiber (HDSSF) via a simple dip-coating method. As shown in Figure 12b, two parallel nylon fibers were immersed in a poly (vinylidene fluoride) (PVDF) solution and withdrawn after solvent evaporation to form spindle-knot structures. The bio-inspired fibers were then subjected to oxygen plasma treatment to impart hydrophilicity. On the HDSSF, a completely different fog water collection mode was observed: under the combined action of capillary force and an internal Laplace pressure gradient, captured droplets were rapidly transported to the spindle-knots along a continuous liquid film. The high fog water collection efficiency of the HDSSF is attributed to the rapid droplet transport through the microchannel between the two parallel threads, the directional droplet collection at the spindle-knots, and the enhanced fog water capture capability of the hydrophilic fibers. Such HDSSFs hold promises for application in high-efficiency fog water collection systems and droplet transport devices.

Most existing spider-silk-inspired fog water collection structures are fabricated from nylon fibers, typically by applying coatings of various polymer solutions onto the nylon via a dip-coating method [140,141]. As shown in Figure 12c, Venkatesan et al. [142] also employed a dip-coating approach to prepare a structure with periodic spindle-knots. Unlike most spindle-knot fabrication methods that use nylon fibers, they formed periodic knots on the surface of degummed silkworm silk fibers by coating them with engineered major ampullate spidroin 2 (eMaSp2), thereby producing an all silk-protein fiber (ASPF). The ASPF is 252 times lighter than the previously reported nylon fiber coated with synthetic polymers that exhibited the highest fog water collection efficiency [143]. Owing to its high density of protein fibers, the ASPF demonstrates a fog water collection efficiency that is 100 times greater than that of other artificial synthetic fibers. The combined effect of the spindle-knot morphology and the hydrophilicity of the protein fibers enables the ASPF to achieve a maximum fog water collection volume of 6.6 µL, more than three times that of untreated fibers without dip-coating (2.2 µL). Given the abundant availability of silkworm silk, its tandem use with recombinant spider silk proteins offers substantial potential for scalability.

The microfluidic method, owing to its advantage of controllable flow rates, has also been employed to fabricate spider-silk-inspired spindle-knot structures. Tian et al. [144] precisely fabricated spindle-knot microfibers using a simple water-in-air microfluidic approach. Fog water collection experiments showed that, for a single spindle-knot microfiber with a length of 7.6 cm, the collected fog water volume increased from 0.1 µL to 2.2641 µL within 600 s. The spindle-knot microfiber exhibited excellent stability, resisting deformation and supporting long-term use (Figure 12d). As shown in Figure 12e, Liu et al. [145] fabricated heterostructure rough spindle-knot microfibers (HRSFs) with a roughness gradient between spindle-knots and joints via a flexible microfluidic method. By adjusting the pump flow rate, the dimensions of the spindle-knots and joints could be precisely controlled. The HRSFs, composed of chitosan and calcium alginate, exhibited excellent mechanical strength and resistance to corrosion in both acidic and alkaline environments. During the fog water collection process, microdroplets deposited and adhered to the surface of the HRSFs, subsequently coalescing and transferring from the joints to the spindle-knot regions. The results demonstrated that the surface morphology of the spindle-knots directly influenced fog water collection efficiency, with a greater roughness gradient between spindle-knots and joints leading to higher efficiency. This microfluidic technique also provides a low-cost and relatively flexible approach for fabricating spider-silk-inspired structures, enabling the production of rough spindle-knot microfibers with high biocompatibility and biomimetic features.

Electrospinning technology, owing to its advantages of tunable fiber diameters and diverse printable materials, has been widely applied in fields such as bioengineering and materials engineering. The aforementioned methods primarily fabricate single-fiber spider-silk-inspired structures, whereas electrospinning can directly produce multi-fiber structures. Du et al. [146] fabricated a novel multi-fiber structured membrane via electrospinning (Figure 12f). Poly (L-lactic acid) (PLLA) was dissolved in chloroform to form a polymer solution, which was injected into the nozzle of the electrospinning apparatus. A voltage of 12 kV was applied between the nozzle and the substrate, enabling fiber membrane formation through electrospinning. Uniformly distributed polymer solution microbeads, mimicking the spindle-knot structures of spider silk, were formed on the fiber membrane. By adjusting the concentration of the polymer solution, microbeads of different shapes could be obtained. Fog water collection experiments revealed that the polymer solution microbeads on the fiber membrane could transport deposited droplets toward the center of the membrane, driven by multiple forces acting on the droplets. Compared with single-fiber structures, this multi-fiber integrated system enables cyclic directional droplet transport, greatly enhancing both the efficiency and continuity of fog water collection. Zhang et al. [147] proposed a dynamic-interface electrospinning process (Figure 12g) in which controllable vibration of the electrospinning nozzle enables precise regulation of spindle-knot size and periodic structure. Under axial stretching, the spindle-knots fabricated by this method also exhibited oriented groove patterns on their surfaces, enhancing the surface energy gradient of the spindle-knots. Fog water collection experiments revealed that the water transport speed was up to 405 times faster than that of natural spider silk, and the droplet-hanging capacity was improved by a factor of 4.7. This fiber structure integrates the spindle-knot morphology of spider silk with the conical geometry of cactus spines, utilizing both surface energy gradients and Laplace pressure to drive the directional motion of small droplets along the fiber surface. In this process, the microstructure and wettability of the material surface play a crucial role: the unique spindle-shaped surface and the resulting surface energy gradient enable droplets to spontaneously migrate toward regions of lower energy. By tuning the material’s wettability, the interaction forces between the fiber surface and droplets can be modulated, thereby influencing droplet motion behavior. Furthermore, by synchronously operating multiple electrospinning nozzles, the authors fabricated microfibers with multi-axis spindle-knot structures; the four-axis spindle-knot configuration exhibited a 1.74-fold improvement in fog water collection performance compared with the original design. The introduction of this electrospinning method and the fabrication of artificial multi-axis spindle-knot fibers represent a significant advancement in the development of spider-silk-inspired fog water collection structures and materials.

Spider silk and spider-silk-inspired biomimetic materials offer several advantages, including low fabrication cost, high controllability of sample properties, and low wind resistance during fog water collection. However, they also exhibit notable limitations. The lack of an effective drainage strategy means that spider silk primarily relies on gravity for water removal, making dispersed droplets difficult to collect. In addition, web-like materials typically have large pore sizes and small surface areas, resulting in high fog-flow transmittance and a low final fog water capture ratio.

### 3.4. Nepenthes mirabilis

*Nepenthes mirabilis* is distributed across tropical continental regions, where its nutrient-poor habitats have driven the evolution of a unique nutrient-absorbing organ known as the “insect-trapping pitcher” [148]. Although *N. mirabilis* does not possess a typical fog water collection capability, the liquid-lubricated layer on its peristome surface and the unidirectional spreading behavior of the surface liquid film provides valuable insights for optimizing droplet transport design following fog water collection.

#### 3.4.1. Droplet Transport Mechanism on the Peristome of *Nepenthes mirabilis*

**Figure 13:**
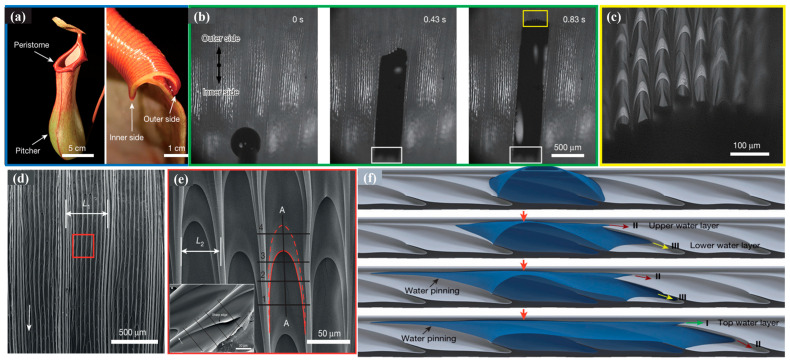
Droplet transport mechanism on the peristome of *Nepenthes mirabilis*. (**a**) Photograph of the *Nepenthes mirabilis* and its peristome region. (**b**) Directional self-transport process of a droplet overcoming gravity on the peristome. (**c**) Magnified photograph of a droplet edge, showing water transport confined within microchannels. (**d**,**e**) Unique secondary microgroove structures on the peristome surface. (**f**) Three-dimensional schematic illustration of directional self-transport of water within the microgrooves (Reprinted with permission from ref. [41], Copyright © 2018 WILEY-VCH Verlag GmbH & Co. KGaA, Weinheim).

The insect-trapping pitcher of *Nepenthes mirabilis* consists of a lid, a peristome, a waxy zone, and a digestive zone. The trichome structures on the lid capture airborne fog droplets, thereby maintaining the slippery, wet condition of the peristome surface [149]. The peristome exhibits superhydrophilicity, enabling the rapid formation of a continuous water film under humid conditions. This film acts as a lubricant, causing insects crawling on it to easily slip into the pitcher, where they are captured and digested (Figure 13a). Inspired by the lubricating effect of the peristome, Chen et al. [41] discovered that droplets deposited on the peristome of *Nepenthes alata* pitchers can overcome gravity and undergo directional spreading from the inner to the outer side of the peristome (Figure 13b,c). Further investigation of the peristome surface of *Nepenthes mirabilis* revealed a distinctive secondary microgroove architecture, in which parallel first-order grooves (L1) contain approximately ten second-order microgrooves each, and within these are periodically arranged, uniformly oriented “duck-billed” wedge-shaped blind cavities with arch-shaped edges (Figure 13d,e). These wedge-shaped cavities possess an arched profile with sharp edges and exhibit a gradient wedge geometry. This hierarchical structure endows the peristome surface with exceptional lubricating properties; the resulting Laplace pressure difference and capillary forces enable uniform spreading of droplets across the peristome. As illustrated in Figure 13f, droplets rapidly ascend within the wedge-shaped grooves until the grooves are filled, after which adjacent grooves are sequentially filled, achieving long-distance directional droplet transport. In addition, the regular hierarchical surface of the pitcher can be fully wetted by nectar secreted from the peristome and by rainwater [149], forming a hydrophilic, smooth liquid-infused porous surface (Figure 13d). Although research on bioinspired liquid-infused porous surfaces mimicking the peristome of *Nepenthes* remains limited, such surfaces hold significant promise for guiding the design of directional droplet transport systems and are expected to attract increasing attention in the future.

#### 3.4.2. Fabrication of Structures for Droplet Transport Inspired by the Peristome of *Nepenthes mirabilis*

**Figure 14:**
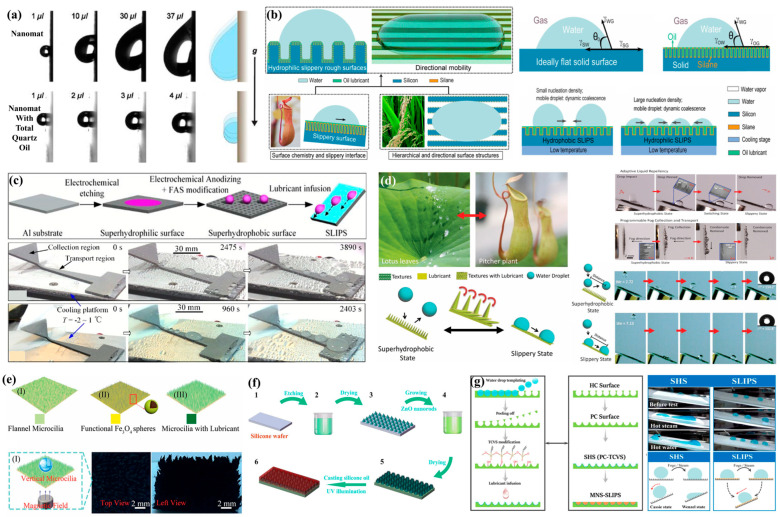
Fabrication of structures for droplet transport inspired by the peristome of Nepenthes mirabilis. (**a**) Photographs showing droplet movement on a nanofiber-net surface infused with silicone oil (Reprinted with permission from ref. [152], Copyright © 2013, American Chemical Society). (**b**) Fog water collection behaviors on superhydrophobic, lubricated, and directionally lubricated surfaces (Reprinted with permission from ref. [153], Copyright © 2018, The American Association for the Advancement of Science). (**c**) Condensation and continuous transport of water on a wedge-shaped collection platform (Reprinted with permission from ref. [154], Copyright © 2013, Royal society of chemistry). (**d**) Application examples of the transformable PDMS/Fe surface. Adaptive liquid repellency and programmable fog water collection and transport (Reprinted with permission from ref. [155], Copyright © 2016 WILEY-VCH Verlag GmbH& Co. KGaA, Weinheim). (**e**) Photographs of the fog water collection and transport processes for the PDMS@Fe3O4 fabric (Reprinted with permission from ref. [156], Copyright © 2013, Royal society of chemistry). (**f**) Schematic illustration of the preparation of a lubricated surface using silicone oil as the lubricant (Reprinted with permission from ref. [157], Copyright © 2019, American Chemical Society). (**g**) Schematic illustration of a bioinspired super-lubricated surface (Reprinted with permission from ref. [158], Copyright © 2020, American Chemical Society).

Inspired by the directional droplet transport observed on the peristome surface of *Nepenthes mirabilis*, slippery liquid-infused porous surfaces (SLIPS) have recently attracted increasing attention. Such surfaces possess numerous advantages, including omnidirectional liquid repellency, self-healing capability, and high optical transparency, and have been widely applied in fields such as self-cleaning coatings, marine antifouling, and biomedical engineering [150,151]. Compared with spider silk, desert beetles, and cactus-inspired designs, the primary advantage of peristome-inspired surfaces lies in their ability to achieve directional droplet transport after fog water collection. Singh et al. [152] infused hydrophobic PVDF-HFP nanofiber mats with silicone oil and perfluoropolyether (Figure 14a) and evaluated the resulting lubricated surfaces in fog water collection tests. The nanofiber mats exhibited a fog water collection efficiency of approximately 118 ± 6 mg·cm^−2^·h^−1^. Singh concluded that liquid-infused slippery surfaces can effectively enhance droplet mobility and transport performance, offering significant value for water vapor harvesting. Following the publication of studies on peristome-inspired surfaces, researchers have increasingly explored the application of such SLIPS designs in fog water collection systems. Dai et al. [153] employed plasma etching to construct a rough nano–microgroove array structure on the surface of a silicon wafer. Following low-surface-energy silane modification and lubricant infusion, the resulting surface enabled controlled directional sliding of droplets and was successfully applied to fog water collection (Figure 14b). On this surface, condensed droplets could be directionally transported, achieving a water collection efficiency of 500 mg·cm^−2^·h^−1^—an improvement over smooth surfaces without a wettability gradient. Luo et al. [154] developed a fog water collection platform capable of both dropwise condensation and dropwise transport. The platform consisted of a wedge-shaped aluminum plate, whose surface was fabricated via electrochemical etching, electrochemical anodization, fluorosilane surface modification, and lubricant infusion. It was found that condensed fog droplets could undergo long-distance, self-driven directional transport in droplet form on this platform (Figure 14c), with a fog water collection efficiency of 61.2 mg·cm^−2^·h^−1^.

In addition, several studies have applied dynamic slippery surfaces to water vapor harvesting. Huang et al. [155] fabricated a magnetically responsive switchable surface capable of transitioning between a superhydrophobic state—similar to that of a lotus leaf—and a slippery liquid-infused porous surface (SLIPS) state resembling the peristome of *Nepenthes mirabilis*. As shown in Figure 14d, this dynamically responsive surface can be maintained in a superhydrophobic state on demand for droplet transport, or in a SLIPS state for fog water collection and transport. Similarly, Li et al. [156] developed a PDMS@Fe_3_O_4_-modified velvety fabric with switchable wettability between SLIPS and a superhydrophobic state, and applied it to fog water collection. By controlling the reversible transition between the two wetting states via a dynamic magnetic field, they were able to regulate both fog droplet condensation and the directional migration of condensed droplets. This fabric achieved a fog water collection efficiency of up to 980 mg·cm^−2^·h^−1^ (Figure 14e). Jing et al. [157] employed a simple hydrothermal method combined with a photocatalytic reaction to graft polydimethylsiloxane (PDMS) onto ZnO nanorods, thereby fabricating a UV-cured structure (Figure 14f). Silicone oil was subsequently infused as a lubricant to form a hierarchical lubricant-impregnated surface (LIS). Owing to the high viscosity of the silicone oil and the strong intermolecular interactions between the silicone oil and PDMS, the lubricant was firmly retained within the micro/nanostructures, resulting in a durable lubricating layer. In fog water collection experiments, the surface maintained excellent directional droplet transport performance, with no evident changes in the lubricant layer. Even after one week of exposure to sunlight, the surface continued to exhibit outstanding droplet directional transport capability.

To further enhance the stability of infused lubricants, Feng et al. [158] developed a highly stable slippery liquid-infused porous surface (SLIPS) by synergistically constructing a regular microspine sponge structure combined with nanoparticles, thereby improving lubricant retention. Notably, the presence of nanoscale features on top of the microstructured surface enhanced capillary forces, enabling the lubricant to be firmly anchored to the surface and reducing lubricant loss (Figure 14g). The fabricated bioinspired SLIPS exhibited excellent fog water collection performance, achieving an efficiency of 852 mg·cm^−2^·h^−1^, and maintained stability during continuous operation for 20 h. In addition, the surface demonstrated long-term usability and high thermal resistance to both steam and hot water. However, lubricant-infused surfaces generally suffer from limited durability, which can lead to a loss of control over droplet motion after repeated use.

Inspired by the peristome surface of *Nepenthes mirabilis*, slippery liquid-infused porous surfaces (SLIPS) have attracted considerable attention since their introduction. Over time, the fabrication and application of SLIPS have gradually matured. Although peristome-inspired surfaces offer numerous advantages in fog water collection and droplet manipulation—such as high collection efficiency, long-distance transport, and reusability—they still present certain limitations. The primary challenges include lubricant loss, reliance on the motion of individual droplets, and difficulty in transporting large-volume droplets. While SLIPS require that the infused lubricant be immiscible with the surface droplets, some lubricant inevitably becomes entrained and removed during droplet motion, potentially contaminating the droplet sample. Furthermore, the hierarchical structures characteristic of peristome-inspired surfaces makes their fabrication processes complex and costly, limiting their suitability for large-scale production. Future work should focus on in-depth mechanistic studies to further optimize their structural design and functional performance.

### 3.5. Multilevel Composite Bioinspired Fog Water Collection Structures

**Figure 15:**
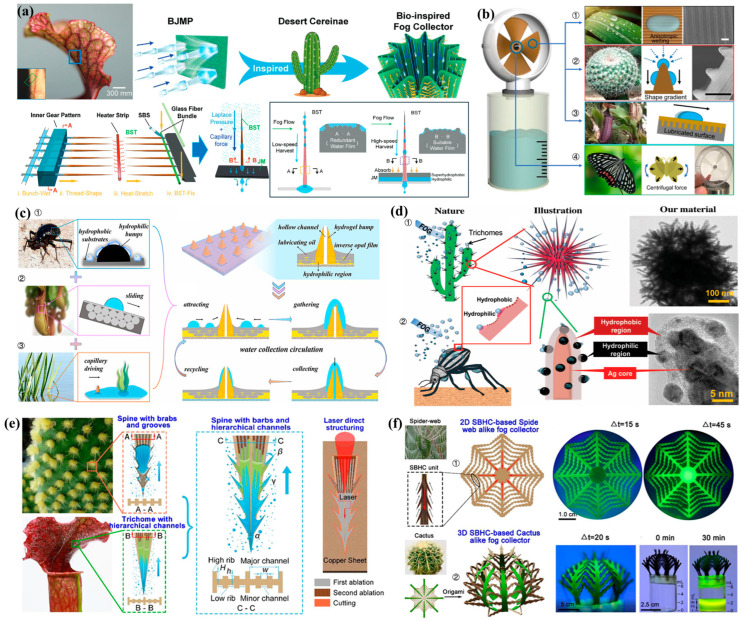
Multilevel composite bioinspired fog water collection structures. (**a**) Schematic illustration of a hierarchical microchannel structure inspired by cactus spines and Nepenthes (Reprinted with permission from ref. [160], Copyright © 2021 The Authors. Global Challenges published by Wiley-VCH GmbH). (**b**) Fog water collection windmill inspired by rice leaves, cactus spines, Nepenthes, and butterflies (Reprinted with permission from ref. [161], Copyright © 2019, American Chemical Society). (**c**) Schematic illustration of a bioinspired multiscale surface structure with hydrophilic hollow convex–concave arrays and its water collection mechanism (Reprinted with permission from ref. [162], Copyright ©2019, National Academy of Sciences). (**d**) Schematic illustration of silver nanowires (NWs) structure with hydrophilic–hydrophobic patterns inspired by cactus spines and the Namib Desert beetle (Reprinted with permission from ref. [163], Copyright © 2018 WILEY-VCH Verlag GmbH& Co. KGaA, Weinheim). (**e**) Schematic illustration of a bioinspired spine structure with backward microbarbs and hierarchical microchannels designed for fog water collection, inspired by cactus spines and Nepenthes (Reprinted with permission from ref. [164], Copyright © 2020, American Chemical Society). (**f**) Fog water collection structures combining a 2D spiderweb-inspired design and a 3D cactus-spine-inspired design, inspired by spider silk and cactus spines (Reprinted with permission from ref. [164], Copyright © 2020, American Chemical Society).

Desert organisms exhibit diverse strategies for fog water collection, each employing distinct mechanisms. Nevertheless, their evolutionary trajectories converge toward a common goal: enhancing fog water collection efficiency by constructing specialized structures or tailoring surface wettability, while simultaneously establishing post-collection droplet transport pathways to minimize evaporation and other losses [159]. In biomimetics, numerous high-performance structures and devices have been developed by emulating these natural organisms (as shown in Table 2). Moreover, the integration of multiple biological features to complement each other’s strengths has emerged as a prominent research focus, aiming to further improve fog water collection performance. As shown in Figure 15a, Chen et al. [160] designed a bionic Sarracenia trichome (BST) with an on-demand regular hierarchical microchannel structure with a peristome-inspired surface on glass fiber bundles via a one-step thermoplastic stretching method. By coupling the BST with a Janus membrane, they constructed a cactus-inspired BST + Janus membrane plate (BJMP) structure. In BST, the primary channels were formed by gear-like patterns, while the secondary microchannels were composed of glass fibers. Coupling with the Janus membrane enhanced capillary condensation and fog water collection performance. The fabricated BJMP established a novel, highly efficient multiscale fog water collection architecture, deliberately incorporating two gradients—surface energy gradient and Laplace pressure gradient. Compared with single-scale structures, this design achieved a threefold improvement in fog water collection efficiency. This low-cost structure is easy to fabricate and offers valuable guidance for fog water collection systems in arid regions. However, while bioinspired fog water collection surfaces perform well under static fog flow conditions, their efficiency decreases when the fog flow direction is variable or under strong wind conditions. To this end, Wang et al. [161] drew inspiration from rice leaves, cacti, *Nepenthes mirabilis*, and butterflies to propose a dynamic fog water collection strategy, as illustrated in Figure 15b. The team developed a windmill-like fog water collection system in which blades with integrated fine-groove structures rotate when driven by wind. During rotation, droplets are deposited or ejected into surrounding volutes upon collision, eventually dripping into a collection container. The bioinspired blade surfaces, featuring integrated fine grooves, not only facilitate efficient fog droplet deposition but also enable directional droplet transport, thereby enhancing water collection efficiency. A key advantage of this design lies in its effective integration of the physicochemical properties of multiple plants and animals with engineering and fabrication considerations. Consequently, the windmill structure can harvest fog water not only under static fog flow conditions but also in variable wind environments, as it can sense wind direction and adjust its rotational speed to harness wind energy for efficient fog water collection. Zhang et al. [162], inspired by the fog water collection strategies of the Namib Desert beetle and the microstructures of *Nepenthes mirabilis*, designed and fabricated a multilayer hierarchical wettability surface (Figure 15c) by combining colloidal self-assembly, photolithography, and templating techniques. The surface consisted of a substrate composed of a hollow hydrogel concave–convex array and a lubricant-infused inverse opal film. Hydrophilic protrusions and hydrophobic membranes mimicked the wettability gradient patterns of the Namib Desert beetle, while the infused lubricant imparted a slippery liquid-infused porous (SLIP) structure analogous to that of *Nepenthes*. The hollow hydrophilic protrusions rapidly aggregated and captured water droplets from all directions via capillary forces, while the highly smooth substrate further facilitated this process. The captured droplets were then pumped downward through the hollow channels of the protrusions under capillary action, ultimately being collected in a reservoir. Inspired by cacti and the Namib Desert beetle, Sarkar et al. [163] developed a high-efficiency fog water collection structure composed of hydrophilic–hydrophobic patterned silver nanowires (NWs) using an ambient ion-based method. This wettability-gradient fog harvester was fabricated via a two-step surface modification of the synthesized NWs using electrospray technology. As shown in Figure 15d, the patterned NWs—approximately 20 µm in length and 200 nm in width, covering an area of up to 2 × 2 cm^2^—exhibited a fog water collection efficiency of 56.6 L·m^−2^·d^−1^. Moreover, the fabrication process for the NWs was straightforward, requiring no large or complex equipment, and could be completed under ambient conditions. In their study, Wang et al. [164] fabricated two types of bioinspired composite structures. The first design was inspired by the conical fog water collection spines with microbarbs of cacti and the trichomes with hierarchical microchannels of *Nepenthes mirabilis* that enable directional droplet transport. Based on these natural models, they developed artificial spines incorporating both backward-facing barbs and layered microchannels. This configuration allowed ultrafast droplet transport and exceptionally high water collection rates. Building on this multifunctional structure, the team further optimized fog water collection efficiency by employing direct laser structuring in combination with origami-based folding techniques to create a second design: a two-dimensional (2D) spider-web-inspired FWCS and a three-dimensional (3D) cactus-inspired FWCS. Experimental evaluations revealed that the 2D spider-web and 3D cactus-like structures achieved fog water collection rates of 5 mL h^−1^ and 5.6 mL h^−1^, respectively (Figure 15f).

At present, the application of bioinspired multilevel structures in fog water collection faces three primary challenges. First, the enhancement mechanisms underlying the improved fog water collection performance of such structures remain a subject of debate. Second, their fabrication is often associated with high costs and poor durability. Third, the device-level implementation of bioinspired multilevel structures is still at an early stage, making large-scale production difficult to achieve.

## 4. Conclusions and Outlook

With the increasing severity of global water scarcity, particularly in arid and semi-arid regions, fog water collection has attracted widespread attention as a low-energy, environmentally friendly technology. In recent years, the design of FWCSs based on bioinspired principles, inspired by the unique structures and mechanisms of plants and animals in nature, has provided new avenues for the advancement of fog water collection technologies. From the precise regulation of surface microstructures to the optimization of multiscale designs, bioinspired structures have demonstrated tremendous potential for enhancing water collection efficiency. This review has summarized the current applications of bioinspired FWCSs, with a particular focus on the underlying physical mechanisms and the latest advances in structural design.

Current research indicates that the fog water collection performance of bioinspired structures depends not only on the design of surface micro- and nanostructures, but is also closely related to factors such as surface wettability, droplet dynamics, and surface energy gradients. Analyses of representative bioinspired materials in nature have revealed how organisms such as the Namib Desert beetle and cactus spines employ specialized structural designs to effectively capture and transport fog water by harnessing physical mechanisms including surface tension, Laplace pressure gradients, and wettability gradients. These insights provide a solid theoretical foundation for the optimization of FWCSs.

Nevertheless, despite the significant progress achieved in recent studies, numerous challenges remain. First, the field deployment of bioinspired FWCSs faces distinct engineering bottlenecks closely tied to natural environmental constraints—issues that have not been fully addressed in current laboratory-based studies. Specifically, fouling problems severely compromise collection efficiency in real-world scenarios: in arid desert regions, sand and dust particles easily accumulate on micro/nanostructures, blocking fog droplet adsorption sites; in coastal fog environments, salt crystal precipitation on surfaces of FWCSs alters surface wettability and disrupts directional droplet transport. Additionally, the long-term stability of chemical coatings and lubricants—core components of many bioinspired designs of FWCSs—may not be guaranteed under harsh field conditions: for example, ultraviolet (UV) radiation-induced coating degradation, lubricant evaporation and loss. Furthermore, cost-effectiveness remains a major barrier to large-scale deployment: high-performance structures rely on precision fabrication techniques; even template-based methods also face challenges in batch production yield and consistency. Second, in terms of structural design, many investigations have focused on a single structure or mechanism, with limited attention to the synergistic optimization of multiscale architectures. Future research could integrate both micro- and macro-scale features through hierarchical design strategies to enhance overall water collection efficiency. Third, although bioinspired structures have demonstrated high fog water collection efficiency under laboratory conditions, ensuring their long-term stability and adaptability in complex natural environments remains an urgent issue. Furthermore, most current bioinspired FWCSs are developed under idealized experimental conditions, and their durability, resistance to wind and sand erosion, and ultraviolet stability still require further verification for practical applications. At present, the lack of standardized testing conditions makes cross-study comparison difficult. Future work should therefore establish unified evaluation metrics and enable meta-analytical approaches to quantitatively assess the fog water collection influence of structure, material, and environmental parameters (such as wind speed, relative humidity and other conditions).

From an application perspective, research on bioinspired FWCSs is not limited to fog water collection but can be extended to diverse fields such as agricultural irrigation, building energy conservation, and environmental remediation. In particular, bioinspired FWCSs hold significant potential in extreme environments such as arid regions, deserts, and mountainous areas. Future studies should consider designing fog water collection systems tailored to the climatic conditions of different regions to enhance their adaptability and efficiency under various environmental scenarios. Moreover, with continuous technological advancements, bioinspired FWCSs are expected to be integrated with other technologies—such as solar cells and energy storage systems—to form interdisciplinary, multifunctional platforms that enable the synergistic utilization of energy and water resources, thereby offering innovative solutions to global water scarcity and environmental protection challenges.

Furthermore, with the continuous advancement of manufacturing technologies—particularly the maturation of 3D printing and nanofabrication—significant breakthroughs are expected in large-scale production and diversified applications. These advanced techniques enable the precise, high-volume fabrication of bioinspired FWCSs while reducing production costs, thereby promoting their widespread deployment in real-world environments. For the long-term maintenance and stability of such structures, smart responsive materials (e.g., shape memory alloys, thermoresponsive materials) [165,166], self-healing technologies [167] and thermal management technologies [168] are anticipated to become prominent research hotspots of fog water collection.

In summary, substantial progress has been made in the design and mechanistic understanding of bioinspired FWCSs. However, translating these advances from laboratory demonstrations to practical applications will require sustained efforts in structural optimization, material selection, fabrication processes, and field validation. Looking ahead, with the emergence of novel materials and fabrication techniques, bioinspired fog water collection technologies are poised for global adoption, offering promising solutions to the escalating challenge of water scarcity and contributing positively to sustainable development.

## Figures and Tables

**Table 1:** Timeline of the development of bioinspired fog water collection structures.

Species Name	Time	Reference
Lotus Leaf	1997	[22]
Rose Petal	2008	[23]
*Stipagrostis sabulicola* (Namibian needle grass)	2012	[24]
Thorny devil (*Moloch horridus*)	2016	[25]
Foxtail Grass	2014	[35]
Butterfly	2007	[36]
Shorebird	2008	[37]
Namib Desert beetle	2001	[38]
Cactus Spines	2012	[39]
Spider Silk	2010	[40]
*Nepenthes mirabilis*	2016	[41]

**Table 2:** Four typical species and experimental conditions.

Species Name	Key Structural Characteristic	The Mechanism of Fog Water Collection	Experiment Condition			FWCSs Efficiency	References
			Wind Speed	Relative Humidity	Size of Fog Droplets		
Namib Desert Beetle	Hydrophilic protrusions on the back + Hydrophobic grooves	Surface energy gradient					[100]
							[101]
			<0.5 m/s	92%			[102]
			3.5 m/s	90%	5–10 μm	1432.7 mg·h^−1^·cm^−2^	[104]
			2.8 m/s	85%			[105]
Cactus Spines	Conical tip + Gradient roughness groove + Bottom strip-like protuberances	Surface energy gradient + Laplace pressure gradient					[116]
							[117]
			3 m/s	85%		0.35 μL/min	[118]
			<0.5 m/s				[119]
			3 m/s	90%	6 μm	2 mg·min^−1^·mm^−3^	[120]
			2 m/s	85%	8–10 μm		[121]
			2.5 m/s	88%	5–8 μm		[122]
Spider Silk	Periodic spindle-knot + Joint structure	Surface energy gradient + Laplace pressure gradient				10.44 μL·h^−1^	[137]
			2.5 m/s	88%	5–8 μm		[139]
			0.3 m/s	95%	3–8 μm	MAX 6.6 μL	[142]
			1.5 m/s	86%			[144]
			2 m/s	85%	6–9 μm		[145]
			2.2 m/s	87%			[146]
			2 m/s	88%			[147]
Nepenthes mirabilis	Surface conical micro-grooves	Surface energy gradient + Laplace pressure gradient	3 m/s	82%	4–9 μm	118 ± 6 mg·cm^−2^·h^−1^	[152]
			2.5 m/s	90%		500 mg·cm^−2^·h^−1^	[153]
			1.8 m/s	85%		61.2 mg·cm^−2^·h^−1^	[154]
			<0.5 m/s				[155]
			3 m/s	87%	5–10 μm	980 mg·cm^−2^·h^−1^	[156]
			2 m/s	86%			[157]
			2.5 m/s	90%	6–8 μm	852 mg·cm^−2^·h^−1^	[158]

## Data Availability

The data are provided within the article.

## Abbreviations

The following abbreviations are used in this review:

FWCS	Fog water collection structure
FWCSs	Fog water collection structures
TiO_2_	titanium dioxide
PS	polystyrene
PTFE	polytetrafluoroethylene
PDMS	polydimethylsiloxane
3D	three dimensional
2D	two dimensional
MNCS	micro/nanostructures
JM	Janus membrane
MPs	magnetic particles
MPAM	magnetic particle-assisted molding
PMMA	Polymethylmethacrylate
SLIPS	slippery liquid-infused porous surfaces
